# Non‐Bivariational Time‐Dependent Orbital‐Quasi‐Optimized Coupled‐Cluster Methods Family for Fermion‐Mixtures Dynamics

**DOI:** 10.1002/jcc.70456

**Published:** 2026-08-05

**Authors:** Haifeng Lang, Takeshi Sato

**Affiliations:** ^1^ Department of Nuclear Engineering and Management, Graduate School of Engineering The University of Tokyo Tokyo Japan; ^2^ Photon Science Center, Graduate School of Engineering The University of Tokyo Tokyo Japan; ^3^ Research Institute for Photon Science and Laser Technology The University of Tokyo Tokyo Japan

**Keywords:** coupled cluster, electronic dynamics, non‐adiabatic dynamics, orbital optimization, vibrational dynamics

## Abstract

Recently, [JCP 161, 114114 (2024)] successfully derived numerically stable and converging (to the time‐dependent complete active space self‐consistent‐field) time‐dependent orthogonal coupled cluster (CC) methods, namely, time‐dependent orbital optimized CC (TD‐ooCC) methods for fermion‐mixtures with arbitrary fermion kinds and numbers, via the time‐dependent bivariational principle and setting constraints in the ansatz. In this work, we extend the TD‐ooCC methods to eighteen non‐bivariational time‐dependent orbital‐quasi‐optimized coupled‐cluster (TD‐oqoCC‐NB) methods. In TD‐oqoCC‐NB methods, the constraints in the ansatz are also used to avoid overparameterization in the complete active space limit and eliminate the numerical instability in the finite truncation case. The equations of motion are not determined by the time‐dependent bivariational principle. Instead, all equations ensuring the convergence to the complete active space limit can be selected flexibly. The applications to electron dynamics and vibrational dynamics are also discussed. In particular, we find that all NB methods violate the Ehrenfest theorem even when they are gauge‐invariant, which distinguishes them from TD‐ooCC methods. The stationary condition of EOMs of each NB methods also provides novel CC methods for time‐independent electronic structure calculations.

## 1 Introduction

Quantitatively simulating the dynamics of fermions and fermion mixtures is a prerequisite for understanding strong‐field and ultrafast phenomena [1, 2, 3, 4, 5, 6, 7, 8, 9, 10]. To bypass the intractable factorial scaling of the exact time‐dependent Schrödinger equation (TDSE), coupled‐cluster [11, 12, 13, 14] (CC) parametrizations, including time‐dependent optimized coupled‐cluster method (TD‐OCC) [15, 16, 17, 18] and orbital‐adapted time‐dependent coupled‐cluster [19] (OATDCC) method, and time‐dependent vibrational coupled cluster with time‐dependent modals (TDMVCC) family [20, 21, 22, 23, 24, 25], time‐dependent orbital‐optimized coupled‐cluster (TD‐ooCC) family [26], have recently gained significant success due to their polynomial scaling and rigorous size‐extensivity. The reduction in scaling from the TDSE to these advanced methods originates from two distinct theoretical steps. The first step introduces dynamical core orbitals (doubly occupied with orbital rotations, labeled with indices $i^{\prime m},j^{\prime m}$, where m labels the fermion kind), active orbitals (arbitrarily occupied, $t^{m},u^{m}$), and virtual orbitals (not occupied, $\alpha^{m},\beta^{m}$) to formulate multiconfiguration time‐dependent Hartree‐Fock [27, 28, 29, 30, 31, 32, 33, 34, 35, 36] (MCTDHF) and time‐dependent complete‐active‐space self‐consistent‐field [37, 38] (TD‐CASSCF) approaches. This limits the full configuration‐interaction expansion to the active space, causing the computational cost to scale factorially only with the active particles. The second step further reduces this cost by employing an exponential parametrization with finite truncations within the active space, thereby achieving true polynomial scaling. In contrast, imposing finite truncations on a linear parametrization within the active space—as seen in time‐dependent multiconfiguration self‐consistent‐field (TD‐MCSCF) method [39, 40, 41], MCTDH [n] [42], multireference MCTDH [n] [43], time‐dependent restricted active space self‐consistent field (TD‐RASSCF) [44, 45, 46] and time‐dependent occupation‐restricted multiple active space (TD‐ORMAS) [46, 47, 48, 49, 50]—also yields polynomial scaling, but critically sacrifices size‐extensivity [12].

Equations‐of‐motion (EOMs) of most CC‐parameterization methods are derived from the time‐dependent bivariational principle [51] (TD‐BIVP) instead of the more common time‐dependent univariational principles in linear parameterization methods, since bra and ket states of CC are not complex conjugates. Reference [51] comprehensively studied TD‐BIVP and CC methods, demonstrating that most of them are based on the complex action, or the real part of the complex action when the parameterization is not complex analytic. As an explicit example, TD‐ooCC methods, which use orthogonal orbitals and systematically converge to TD‐CASSCF for arbitrary fermion mixtures, are derived from the TD‐BIVP based on the real part of the complex action. It is also pointed out that TD‐BIVP based on the imaginary part of the complex action is equally valid when the manifold is not “diagonal” [51]. Nonetheless, this is not the case for the TD‐ooCC analog, and potential difficulties exist due to the orthogonal active orbital space in the ansatz. The possible alleviation strategies for the difficulty are one of our main focuses of this work. From a mathematical point of view, the difficulty arises from the incompatibility of the imaginary part of the complex action and orbital rotations other than active‐active orbital rotations. Therefore, one can keep only “valid EOMs” from imaginary part action and replace “invalid EOMs” with those derived from the real part action. Heuristically speaking, this replacement violates the TD‐BIVP but keep the most structures of TD‐BIVP imaginary part action. For this reason, the methods are named as non‐bivariational time‐dependent orbital‐quasi‐optimized coupled‐cluster (TD‐oqoCC‐NB, or NB methods for simplicity) methods. Motivated by this replacement, we also proposed many other TD‐oqoCC‐NB methods which completely disobey TD‐BIVP for “invalid EOMs.” It is important to emphasize that the disobeyance of TD‐BIVP is not necessarily a disadvantage; for instance, restricted polar TDMVCC (rpTDMVCC) [24] is one of the successful CC methods for vibrational dynamics, although it disobeys TD‐BIVP. In fact, the disobeyance can lead to some practical advantages in certain cases, for instance, Reference [46] reported a method using weighted EOMs of Lagrangian [52] and McLachlan variational principle [53] leading to more stable propagation without harming accuracy in strong‐field dynamics. This work will analyze properties of NB methods on the application to vibrational and electron dynamics in detail. We stress that the present work focuses on a comprehensive theoretical analysis of the necessary properties and important modifications on observable evaluations for each application scenario of these methods. Therefore, a valuable list tailored to each scenario guiding future numerical benchmarking, instead of definitive recommendations regarding the optimal choice of methods, is provided.

This paper is organized as follows. In Section 2, we first review the TD‐ooCC methods [26] briefly but with all important features, including the selection of constraints, EOMs, and non‐single (de)excitations. Further, the geometric framework of TD‐BIVP [51] is reviewed. In particular, the degenerate manifold problem [51] in the TD‐BIVP using imaginary part action is explicitly demonstrated via the active‐virtual orbital rotations of the TD‐ooCC. In Section 3, we first derived the imaginary part action‐TD‐BIVP analogs of TD‐ooCC, which is justified in the case of the absence of virtual orbitals and dynamical cores. Based on EOMs of TD‐ooCC methods and their analogs, we formulated eighteen TD‐oqoCC‐NB methods via selecting constraints, EOMs, and non‐single (de)excitations flexibly without satisfying TD‐BIVP. We also discuss major applications of NB methods to vibrational dynamics and electronic dynamics. In particular, the gauge invariance and Ehrenfest theorem are analyzed in detail. Section 4 summarizes our theories and provides an outlook for future work. The Hartree atomic units are used throughout this paper.

## 2 Review

In this section, we will review the framework of TD‐ooCC and TD‐BIVP. They serve as the foundation of TD‐oqoCC‐NB methods.

### 2.1 Review of the TD‐ooCC Family

In a nutshell, CC‐ansatz including all particle‐hole orbital rotations, single excitations, and single deexcitations is redundant in the CAS limit, and numerically unstable at finite truncations. TD‐ooCC methods resolve this problem via regarding at least one of these variables as constraints and setting as zero (equivalently remove them in the ansatz). EOMs are obtained by the TD‐BIVP using the real part of the action. We adopt the notation for orbitals and CC excitations from our previous work Reference [26]. The coupled‐cluster Lagrangian L and the corresponding real action functional $S_{R}$ with an arbitrary Hamiltonian Ĥ with particle conservation [12, 15, 19, 26, 54] can be expressed as,

(1) $$\begin{array}{ll} L & =\langle \Psi_{\text{L}}|(Ĥ-i\partial_{t})|\Psi_{\text{R}}\rangle \;,\; \end{array}$$

(2) $$\begin{array}{ll} S_{R} & =ℜ\int_{t_{0}}^{t_{1}}Ldt=\frac{1}{2}\int_{t_{0}}^{t_{1}}\left(L+L^{*}\right)dt\;.\; \end{array}$$

EOMs of parameters are given by the stationary condition of the action with respect to the variation of all parameters of left‐ and right‐state wavefunctions (with necessary constraints),

(3) $$\begin{array}{c} \begin{array}{ll} 2\delta S_{R} & =\delta \tau_{\overset{˚}{I}}^{\overset{˚}{A}}\left\{\langle \Psi_{\text{L}}|[Ĥ-i\hat{X},{Ê}_{\overset{˚}{I}}^{\overset{˚}{A}}]|\Psi_{\text{R}}\rangle +i{\dot{\lambda }}_{\overset{˚}{A}}^{\overset{˚}{I}}\right\}\\ & \;+\delta \lambda_{\overset{˚}{A}}^{\overset{˚}{I}}\left\{\langle \Phi_{\overset{˚}{I}}^{\overset{˚}{A}}|e^{-\hat{T}}(Ĥ-i\hat{X})|\Psi_{\text{R}}\rangle -i{\dot{\tau }}_{\overset{˚}{I}}^{\overset{˚}{A}}\right\}+c.c\\ & \;+\underset{m}{\sum }\Delta_{\nu^{m}}^{\mu^{m}}\left\{\left\langle \Psi_{\text{L}}|[Ĥ-i(\partial_{t}^{\prime }\hat{T})-i\hat{X},{Ê}_{\nu^{m}}^{\mu^{m}}]|\Psi_{\text{R}}\right\rangle \right.\\ & \;+i\langle \Phi |(\partial_{t}^{\prime }\hat{\Lambda })e^{-\hat{T}}{Ê}_{\nu^{m}}^{\mu^{m}}|\Psi_{\text{R}}\rangle \;\\ & \;-{\left\langle \Psi_{\text{L}}|[Ĥ-i(\partial_{t}^{\prime }\hat{T})-i\hat{X},{Ê}_{\mu^{m}}^{\nu^{m}}]|\Psi_{\text{R}}\right\rangle }^{*}\\ & \;\left.+i{\left\langle \Phi |(\partial_{t}^{\prime }\hat{\Lambda })e^{-\hat{T}}{Ê}_{\mu^{m}}^{\nu^{m}}|\Psi_{\text{R}}\right\rangle }^{*}\right\}\;. \end{array} \end{array}$$

Here, $\mu^{m}$ and $\nu^{m}$ represent general time‐dependent orbitals, and $X_{\nu^{m}}^{\mu^{m}}=\langle \psi_{\mu^{m}}|{\dot{\psi }}_{\nu^{m}}\rangle$. More orbital labels are summarized in Table 1, as required for a complete description of methods. In particular, active orbitals can be further divided into hole orbitals ($i^{m},j^{m},k^{m}$) and particle orbitals ($a^{m},b^{m},c^{m}$), and EOMs of frozen‐core orbitals ($i^{\prime^{\prime }m},j^{\prime^{\prime }m}$) are decided by the pre‐determined constraints ($X_{i^{\prime^{\prime }m}}^{\mu^{m}}$) rather than TD‐BIVP. The CC wave functions are parameterized as $\left|\Psi_{\text{R}}\rangle =e^{\hat{T}}|\Phi \right\rangle$ and $\langle \Psi_{\text{L}}|=\langle \Phi |\hat{\Lambda }e^{-\hat{T}}$, where $\hat{T}=\tau_{\overset{˚}{I}}^{\overset{˚}{A}}{Ê}_{\overset{˚}{I}}^{\overset{˚}{A}}=\sum_{m}\tau_{i^{m}}^{a^{m}}{Ê}_{i^{m}}^{a^{m}}+\tau_{I}^{A}{Ê}_{I}^{A}\;,\hat{\Lambda }=\lambda_{\overset{˚}{A}}^{\overset{˚}{I}}{Ê}_{\overset{˚}{A}}^{\overset{˚}{I}}=\sum_{m}\lambda_{a^{m}}^{i^{m}}{Ê}_{a^{m}}^{i^{m}}+\lambda_{A}^{I}{Ê}_{A}^{I}$ (Einstein notation is always used in this work except for the fermion kind label m), and |Φ⟩ is the reference configuration. General hole and particle strings are represented by $\overset{˚}{I}=ijk\text{⋯}$ and $\overset{˚}{A}=abc\text{⋯}$ (can be empty), and it can further decompose into single and non‐single strings (denoted by I and A.) The general‐rank excitation (deexcitation) operators are ${Ê}_{\overset{˚}{I}}^{\overset{˚}{A}}$ (${Ê}_{\overset{˚}{A}}^{\overset{˚}{I}}$) with corresponding amplitudes $\tau_{\overset{˚}{I}}^{\overset{˚}{A}}$ ($\lambda_{\overset{˚}{A}}^{\overset{˚}{I}}$). Explicitly, ${Ê}_{\nu_{1}^{m_{1}}\nu_{2}^{m_{2}}\nu_{3}^{m_{3}}\text{⋯}}^{\mu_{1}^{m_{1}}\mu_{2}^{m_{2}}\mu_{3}^{m_{3}}\text{⋯}}={ĉ}_{\mu_{1}^{m_{1}}}^{†}{ĉ}_{\mu_{2}^{m_{2}}}^{†}{ĉ}_{\mu_{3}^{m_{3}}}^{†}\text{⋯}{ĉ}_{\nu_{3}^{m_{3}}}{ĉ}_{\nu_{2}^{m_{2}}}{ĉ}_{\nu_{1}^{m_{1}}}$, reducing to ${Ê}_{0}=Î$ for the zero‐rank excitation (empty strings of $\overset{˚}{I}$ and $\overset{˚}{A}$). We also introduce notations $\partial_{t}^{\prime }\hat{T}={\dot{\tau }}_{\overset{˚}{I}}^{\overset{˚}{A}}{Ê}_{\overset{˚}{I}}^{\overset{˚}{A}}$, $\partial_{t}^{\prime }\hat{\Lambda }={\dot{\lambda }}_{\overset{˚}{A}}^{\overset{˚}{I}}{Ê}_{\overset{˚}{A}}^{\overset{˚}{I}}$, $\hat{X}=\sum_{m}X_{\nu^{m}}^{\mu^{m}}{Ê}_{\nu^{m}}^{\mu^{m}}$, $\hat{\Delta }=\sum_{m}\Delta_{\nu^{m}}^{\mu^{m}}{Ê}_{\nu^{m}}^{\mu^{m}}$, $\langle \Phi_{\overset{˚}{I}}^{\overset{˚}{A}}|=\langle \Phi |{Ê}_{\overset{˚}{A}}^{\overset{˚}{I}}$, and $\Delta_{\nu^{m}}^{\mu^{m}}=\langle \psi_{\mu^{m}}|\delta \psi_{\nu^{m}}\rangle$.

**TABLE 1:** Table of the orbital notations and classifications.

Orbital types	Orbital notations	Subclassifications of the orbital^a^
General time‐dependent orbitals	$\mu^{m},\nu^{m},\gamma^{m},\delta^{m}$	$\alpha^{m},p^{m},i^{\prime^{\prime }m}$
Virtual orbitals	$\alpha^{m},\beta^{m}$	——^b^
Frozen‐core orbitals	$i^{\prime^{\prime }m},j^{\prime^{\prime }m}$	——
Occupied orbitals other than frozen‐core orbitals	$p^{m},q^{m},r^{m}$	$i^{\prime m},t^{m}$
Dynamical cores	$i^{\prime m},j^{\prime m},k^{\prime m}$	——^b^
Active orbitals	$t^{m},u^{m}$	$i^{m},a^{m}$
Hole orbitals	$i^{m},j^{m},k^{m}$	——^b^
Particle orbitals	$a^{m},b^{m},c^{m}$	——^b^

^a^ The subclassifications do not list the subclass of the subclass of each type of orbitals.

^b^ “——” represents that the orbitals cannot be further classified.

$X_{\nu^{m}}^{\mu^{m}}$ and $\Delta_{\nu^{m}}^{\mu^{m}}$ are anti‐Hermitian to ensure the orthonormality of orbitals. Additionally, $\Delta_{i^{\prime \prime m}}^{\mu^{m}}\equiv 0$ due to the fact that EOMs of frozen‐core orbitals are determined by pre‐determined constraints. Carefully choosing the non‐single (de)excitations, hole‐hole and particle‐particle rotation invariance of TD‐ooCC methods can be satisfied, see discussions later in this subsection. Additionally, TD‐ooCC methods (as well as NB methods) satisfy the dynamical‐cores‐dynamical‐cores and virtual‐virtual rotation invariance automatically. We set $X_{j^{\prime m}}^{i^{\prime m}}$, $X_{i^{m}}^{j^{m}}$, $X_{a^{m}}^{b^{m}}$, and $X_{\alpha^{m}}^{\beta^{m}}$ as zero to remove the redundancy induced by the rotation invariance. We also use the same constraints for NB methods.

TD‐ooCC methods are derived from the TD‐BIVP, and different constraints selections (single (de)exctations and particle‐hole (ph) rotations) result in the different TD‐ooCC methods. All methods share the same EOMs of non‐single CC amplitudes [Equations (5) and (6)] and orbital rotations other than active‐active, $X_{p^{m}}^{\alpha^{m}}$ and $X_{i^{\prime m}}^{t^{m}}$, which are obtained from the variation with respect to $\Delta_{\alpha^{m}}^{p^{m}}$ and $\Delta_{t^{m}}^{i^{\prime m}}$,

(4) $$\begin{array}{cc} & \langle \Psi_{\text{L}}|[Ĥ-i\hat{X},{Ê}_{\alpha^{m}}^{p^{m}}]|\Psi_{\text{R}}\rangle +\langle \Psi_{\text{R}}|[Ĥ-i\hat{X},{Ê}_{\alpha^{m}}^{p^{m}}]|\Psi_{\text{L}}\rangle =0\;,\\ & \langle \Psi_{\text{L}}|[Ĥ-i\hat{X},{Ê}_{t^{m}}^{i^{\prime m}}]|\Psi_{\text{R}}\rangle +\langle \Psi_{\text{R}}|[Ĥ-i\hat{X},{Ê}_{t^{m}}^{i^{\prime m}}]|\Psi_{\text{L}}\rangle =0\;. \end{array}$$

More practical EOMs of orbital rotations Equation (A1) for simulations will be presented in the Appendix A. Variations with respect to non‐single (zero, two or more particle excitation and deexcitation) CC amplitudes, $\delta S/\delta \lambda_{A}^{I}=0$ and $\delta S/\delta \tau_{I}^{A}=0$ give EOMs of non‐single CC amplitudes,

(5) $$\begin{array}{ll} i{\dot{\tau }}_{I}^{A} & =\langle \Phi_{I}^{A}|e^{-\hat{T}}(Ĥ-i\hat{X})|\Psi_{\text{R}}\rangle \;,\; \end{array}$$

(6) $$\begin{array}{ll} -i{\dot{\lambda }}_{A}^{I} & =\langle \Psi_{\text{L}}|[Ĥ-i\hat{X},{Ê}_{I}^{A}]|\Psi_{\text{R}}\rangle \;.\; \end{array}$$

We select non‐single excitations that satisfy the property

(7) $$\begin{array}{cc} & {Ê}_{\nu^{m}}^{\mu^{m}}|\Psi_{\text{R}}\rangle =e^{\hat{T}}\hat{\Pi }e^{-\hat{T}}{Ê}_{\nu^{m}}^{\mu^{m}}|\Psi_{\text{R}}\rangle \;,\\ & \langle \Psi_{\text{L}}|{Ê}_{\nu^{m}}^{\mu^{m}}=\langle \Psi_{\text{L}}|{Ê}_{\nu^{m}}^{\mu^{m}}e^{\hat{T}}\hat{\Pi }e^{-\hat{T}}\;, \end{array}$$

where $\hat{\Pi }=|\Phi_{\overset{˚}{I}}^{\overset{˚}{A}}\rangle \langle \Phi_{\overset{˚}{I}}^{\overset{˚}{A}}|$, which is sufficient to ensure the hole‐hole (hh) and particle‐particle (pp) rotation invariance for all members in the TD‐ooCC family. Here $\left\{\mu^{m},\nu^{m}\}\in \{i^{m},j^{m}\right\}$ or $\left\{a^{m},b^{m}\right\}$. The simplest examples are D/DT/DTQ/… truncations, and more sophisticated truncations include the weighted excitation truncation [25, 26, 55] and group excitation truncation [26], for details, see section 2.C.2 of Reference [26].

Similar to the non‐single excitations, variation with respect to single (one particle excitation and deexcitation) CC amplitudes give

(8) $$\begin{array}{ll} i{\dot{\tau }}_{i^{m}}^{a^{m}} & =\langle \Phi_{i^{m}}^{a^{m}}|e^{-\hat{T}}(Ĥ-i\hat{X})|\Psi_{\text{R}}\rangle \;,\; \end{array}$$

(9) $$\begin{array}{ll} -i{\dot{\lambda }}_{a^{m}}^{i^{m}} & =\langle \Psi_{\text{L}}|[Ĥ-i\hat{X},{Ê}_{i^{m}}^{a^{m}}]|\Psi_{\text{R}}\rangle \;.\; \end{array}$$

The variation of $\delta S_{R}$ with respect to $\Delta_{a^{m}}^{i^{m}}$ gives

(10) $$\begin{array}{cc} & \;\langle \Psi_{\text{L}}|[Ĥ-i\hat{X}-i(\partial_{t}^{\prime }\hat{T}),{Ê}_{a^{m}}^{i^{m}}]|\Psi_{\text{R}}\rangle \\ + & i\langle \Phi |(\partial_{t}^{\prime }\hat{\Lambda })e^{-\hat{T}}{Ê}_{a^{m}}^{i^{m}}|\Psi_{\text{R}}\rangle \\ - & {\left\langle \Psi_{\text{L}}|[Ĥ-i\hat{X}-i(\partial_{t}^{\prime }\hat{T}),{Ê}_{i^{m}}^{a^{m}}]|\Psi_{\text{R}}\right\rangle }^{*}\\ + & i{\left\langle \Phi |(\partial_{t}^{\prime }\hat{\Lambda })e^{-\hat{T}}{Ê}_{i^{m}}^{a^{m}}|\Psi_{\text{R}}\right\rangle }^{*}=0\;, \end{array}$$

Substituting Equations (5) and (6) into Equation (10) yields,

(11) $$\begin{array}{cc} & \frac{i}{2}(\delta_{b^{m}}^{a^{m}}\rho_{i^{m}}^{j^{m}}-\rho_{b^{m}}^{a^{m}}\delta_{i^{m}}^{j^{m}}){\dot{\tau }}_{j^{m}}^{b^{m}}\\ & \;+i(\delta_{b^{m}}^{a^{m}}D_{i^{m}}^{j^{m}}-D_{b^{m}}^{a^{m}}\delta_{i^{m}}^{j^{m}})X_{j^{m}}^{b^{m}}+i\frac{1}{2}{\left({\dot{\lambda }}_{a^{m}}^{i^{m}}\right)}^{*}=\\ & B_{i^{m}}^{a^{m}}-i\frac{1}{2}\underset{n}{\sum }\left\{\langle \Phi_{j^{n}}^{b^{n}}|e^{-\hat{T}}{Ê}_{a^{m}}^{i^{m}}|\Psi_{\text{R}}\rangle {\dot{\lambda }}_{b^{n}}^{j^{n}}+A_{i^{m}b^{n}}^{a^{m}j^{n}}{\left(X_{j^{n}}^{b^{n}}\right)}^{*}\right\}\;, \end{array}$$

where

(12) $$\begin{array}{ll} A_{i^{m}b^{n}}^{a^{m}j^{n}} & =\langle \Psi_{\text{L}}|[{Ê}_{I}^{A},{Ê}_{b^{n}}^{j^{n}}]|\Psi_{\text{R}}\rangle \langle \Phi_{I}^{A}|e^{-\hat{T}}{Ê}_{a^{m}}^{i^{m}}|\Psi_{\text{R}}\rangle \\ & \;-\langle \Psi_{\text{L}}|[{Ê}_{I}^{A},{Ê}_{a^{m}}^{i^{m}}]|\Psi_{\text{R}}\rangle \langle \Phi_{I}^{A}|e^{-\hat{T}}{Ê}_{b^{n}}^{j^{n}}|\Psi_{\text{R}}\rangle ,\; \end{array}$$

(13) $$\begin{array}{c} \begin{array}{ll} B_{i^{m}}^{a^{m}} & =-\frac{1}{2}\langle \Psi_{\text{L}}|[Ĥ,{Ê}_{a^{m}}^{i^{m}}]|\Psi_{\text{R}}\rangle -\frac{1}{2}\langle \Psi_{\text{R}}|[Ĥ,{Ê}_{a^{m}}^{i^{m}}]|\Psi_{\text{L}}\rangle \\ & \;-\frac{1}{2}\langle \Psi_{\text{L}}|[{Ê}_{I}^{A},Ĥ]|\Psi_{\text{R}}\rangle \langle \Phi_{I}^{A}|e^{-\hat{T}}{Ê}_{a^{m}}^{i^{m}}|\Psi_{\text{R}}\rangle \\ & \;+\frac{1}{2}\langle \Psi_{\text{L}}|[{Ê}_{I}^{A},{Ê}_{a^{m}}^{i^{m}}]|\Psi_{\text{R}}\rangle \langle \Phi_{I}^{A}|e^{-\hat{T}}Ĥ|\Psi_{\text{R}}\rangle \;,\; \end{array} \end{array}$$

(14) $$\begin{array}{ll} \rho_{q^{m}}^{p^{m}} & =\langle \Psi_{\text{L}}|{Ê}_{p^{m}}^{q^{m}}|\Psi_{\text{R}}\rangle \;,D_{q^{m}}^{p^{m}}=\frac{1}{2}(\rho_{q^{m}}^{p^{m}}+{\left(\rho_{p^{m}}^{q^{m}}\right)}^{*})\;.\; \end{array}$$

Equations ((8), (9), (11)) are the starting point of the main discussions of TD‐ooCC methods [26]. Setting corresponding constraints in Equations ((8), (9), (11)), EOMs of single amplitudes (${\dot{\tau }}_{i^{m}}^{a^{m}}$, ${\dot{\lambda }}_{a^{m}}^{i^{m}}$) and particle‐hole orbital rotations ($X_{i^{m}}^{a^{m}}$) can be obtained, which will be listed in the following. If we set different constraints for different fermion kinds, we get the TD‐OCCH, for details, see the Appendix of Reference [26]. The constraints are crucial for TD‐ooCC methods, which avoid numerical instability at finite CC truncations and eliminate redundancy (overparameterization) in the CAS limit. TD‐OCCT1, TD‐BCC, and TD‐OCCX0 can converge to the TD‐CASSCF. It is important to emphasize that constraints of $\lambda_{a^{m}}^{i^{m}}$, $\tau_{i^{m}}^{a^{m}}$, and $X_{i^{m}}^{a^{m}}$ effectively remove Equations (8), (9), and (11), respectively, because of the TD‐BIVP, that is, once the constraints are fixed, ansatz as well as EOMs are automatically determined. From this point of view, listing all EOMs before setting the constraints provides theoretical insights into TD‐ooCC, though it is not necessary to derive EOMs, which is significantly different from NB methods. For details, see Section 3.2.

#### 2.1.1 TD‐OCC

TD‐OCC was first formulated in Reference [15] for electron dynamics, in Reference [21] for vibrational dynamics (named as oTDMVCC), and extended to arbitrary fermion mixtures and identified as a member of TD‐ooCC in Reference [26]. It is the time‐dependent extensions of stationary OCC [56, 57, 58, 59]. All single CC amplitudes are excluded in the ansatz, $\tau_{i^{m}}^{a^{m}}=\lambda_{a^{m}}^{i^{m}}\equiv 0$, and EOMs are from Equation (11),

(15) $$i(\delta_{b^{m}}^{a^{m}}D_{i^{m}}^{j^{m}}-D_{b^{m}}^{a^{m}}\delta_{i^{m}}^{j^{m}})X_{j^{m}}^{b^{m}}+i\underset{n}{\sum }\frac{1}{2}A_{i^{m}b^{n}}^{a^{m}j^{n}}{\left(X_{j^{n}}^{b^{n}}\right)}^{*}=B_{i^{m}}^{a^{m}}.$$

#### 2.1.2 TD‐OCCT1

TD‐OCCT1 excludes single deexcitation in the ansatz, $\lambda_{a^{m}}^{i^{m}}\equiv 0$, and EOMs are from Equations (9) and (11),

(16) $$i\left(\rho_{j^{m}}^{i^{m}}\delta_{b^{m}}^{a^{m}}-\rho_{a^{m}}^{b^{m}}\delta_{j^{m}}^{i^{m}}\right)X_{b^{m}}^{j^{m}}=\langle \Psi_{\text{L}}|[Ĥ,{Ê}_{i^{m}}^{a^{m}}]|\Psi_{\text{R}}\rangle ,$$

(17) $$\begin{array}{cc} & \frac{i}{2}(\delta_{b^{m}}^{a^{m}}\rho_{i^{m}}^{j^{m}}-\rho_{b^{m}}^{a^{m}}\delta_{i^{m}}^{j^{m}}){\dot{\tau }}_{j^{m}}^{b^{m}}=\\ & B_{i^{m}}^{a^{m}}-i(\delta_{b^{m}}^{a^{m}}D_{i^{m}}^{j^{m}}-D_{b^{m}}^{a^{m}}\delta_{i^{m}}^{j^{m}})X_{j^{m}}^{b^{m}}-i\underset{n}{\sum }\frac{1}{2}A_{i^{m}b^{n}}^{a^{m}j^{n}}{\left(X_{j^{n}}^{b^{n}}\right)}^{*}\;. \end{array}$$

Reference [26] proved that TD‐OCCT1 is, up to different $X_{i^{m}}^{j^{m}}$ and $X_{a^{m}}^{b^{m}}$ constraints, mathematically equivalent to sOATDCC (or sTDMVCC) [22] for electron (or vibrational) dynamics, which uses bi‐orthogonal active orbitals spanning the same active space without single excitations and deexcitations. The difference between TD‐OCCT1 and sOATDCC in the orbital rotation constraints and orthogonal/bi‐orthogonal orbitals may make methods perform differently at finite arithmetic. TD‐OCCT1 can be considered as an extension of nonorthogonal orbital optimized coupled cluster (NOCC) [60] with virtual orbitals and dynamical cores.

#### 2.1.3 TD‐BCC

Time‐dependent Brueckner coupled cluster (TD‐BCC) excludes single excitation in the ansatz, $\tau_{i^{m}}^{a^{m}}\equiv 0$, and EOMs are from Equations (8) and (11),

(18) $$\begin{array}{ll} iX_{i^{m}}^{a^{m}}+\underset{n}{\sum }i & \langle \Phi_{i^{m}}^{a^{m}}|e^{-\hat{T}}{Ê}_{b^{n}}^{j^{n}}|\Psi_{\text{R}}\rangle X_{b^{n}}^{j^{n}}=\langle \Phi_{i^{m}}^{a^{m}}|e^{-\hat{T}}Ĥ|\Psi_{\text{R}}\rangle \;,\; \end{array}$$

(19) $$\begin{array}{ll} & i\frac{1}{2}\left\{\underset{n}{\sum }\langle \Phi_{j^{n}}^{b^{n}}|e^{-\hat{T}}{Ê}_{a^{m}}^{i^{m}}|\Psi_{\text{R}}\rangle {\dot{\lambda }}_{b^{n}}^{j^{n}}+{\left({\dot{\lambda }}_{a^{m}}^{i^{m}}\right)}^{*}\right\}=\\ & B_{i^{m}}^{a^{m}}-i(\delta_{b^{m}}^{a^{m}}D_{i^{m}}^{j^{m}}-D_{b^{m}}^{a^{m}}\delta_{i^{m}}^{j^{m}})X_{j^{m}}^{b^{m}}-i\underset{n}{\sum }\frac{1}{2}A_{i^{m}b^{n}}^{a^{m}j^{n}}{\left(X_{j^{n}}^{b^{n}}\right)}^{*}\;,\; \end{array}$$

which can be considered as a time‐dependent extension of the BCC [61, 62, 63, 64].

#### 2.1.4 TD‐OCCX0

TD‐OCCX0 excludes particle‐hole orbital rotations, and EOMs of the single amplitudes of TD‐OCCX0 are given by Equations (8) and (9). It is a direct extension of TD‐CC with virtual orbitals and dynamical cores. The major difference between TD‐OCCX0 and other TD‐ooCC methods is that the former one does not obey the Ehrenfest theorem, due to the lack of invariance under particle‐hole orbital rotations. The detailed formulation of the modified Ehrenfest theorem will be discussed in Section 3.5.

### 2.2 Review of TD‐BIVP

Recently, Reference [51] studied TD‐BIVP from a differential geometric point of view and proposed two distinct TD‐BIVP for real bivariational approximation submanifolds. In a nutshell, the TD‐BIVP based on the complex action $\delta \int_{t_{0}}^{t_{1}}Ldt=0$ is ill‐defined if complex differentiability does not hold. In this case, the TD‐BIVP can be defined according to the real manifolds, leading to the TD‐BIVP based on the real part of the complex action $\delta ℜ\int_{t_{0}}^{t_{1}}Ldt=0$ and/or on the imaginary part of the complex action $\delta ℑ\int_{t_{0}}^{t_{1}}Ldt=0$. $\delta ℜ\int_{t_{0}}^{t_{1}}Ldt=0$ and $\delta ℑ\int_{t_{0}}^{t_{1}}Ldt=0$ are equivalent if $\delta \int_{t_{0}}^{t_{1}}Ldt=0$ is well‐defined. The relationship between $\delta \int_{t_{0}}^{t_{1}}Ldt=0$, $\delta ℜ\int_{t_{0}}^{t_{1}}Ldt=0$, and $\delta ℑ\int_{t_{0}}^{t_{1}}Ldt=0$ in TD‐BIVP is analogous to that among the Dirac‐Frenkel principle, the time‐dependent variational principle, and the McLachlan principle in time‐dependent univariational principles. Furthermore, the time‐dependent univariational principles can be reconstructed from the TD‐BIVP.

Most CC‐based methods [51] are based on the complex action or the real part of the action, for instance, TD‐ooCC is based on the real part of the action. We emphasize that the real part of the action for TD‐ooCC is necessary since its parameterization is not complex analytic. Consequently, one might naively expect that valid EOMs could also be derived for the TD‐ooCC ansatz using the imaginary part of the action. However, it is important to emphasize that the manifold of $\delta ℑ\int_{t_{0}}^{t_{1}}Ldt=0$ can be degenerate when the manifold can be reduced to the “diagonal” [51], leading to meaningless, or invalid EOMs. As a concrete example, we consider TD‐ooCC‐type ansatz with imaginary action

(20) $$S_{I}=ℑ\int_{t_{0}}^{t_{1}}Ldt=\frac{1}{2i}\int_{t_{0}}^{t_{1}}\left(L-L^{*}\right)dt,$$

EOMs of intergroup rotations $X_{p^{m}}^{\alpha^{m}}$ and $X_{i^{\prime m}}^{t^{m}}$ are obtained from the variation with respect to $\Delta_{\alpha^{m}}^{p^{m}}$ and $\Delta_{t^{m}}^{i^{\prime m}}$,

(21) $$\begin{array}{cc} & \langle \Psi_{\text{L}}|[Ĥ-i\hat{X},{Ê}_{\alpha^{m}}^{p^{m}}]|\Psi_{\text{R}}\rangle -\langle \Psi_{\text{R}}|[Ĥ-i\hat{X},{Ê}_{\alpha^{m}}^{p^{m}}]|\Psi_{\text{L}}\rangle =0\;,\\ & \langle \Psi_{\text{L}}|[Ĥ-i\hat{X},{Ê}_{t^{m}}^{i^{\prime m}}]|\Psi_{\text{R}}\rangle -\langle \Psi_{\text{R}}|[Ĥ-i\hat{X},{Ê}_{t^{m}}^{i^{\prime m}}]|\Psi_{\text{L}}\rangle =0\;, \end{array}$$

which can be simplified to

(22) $$\begin{array}{cc} & -\langle \Psi_{\text{L}}|{Ê}_{\alpha^{m}}^{p^{m}}(Ĥ-i\hat{X})|\Psi_{\text{R}}\rangle +\langle \Psi_{\text{R}}|{Ê}_{\alpha^{m}}^{p^{m}}(Ĥ-i\hat{X})|\Psi_{\text{L}}\rangle =0\;,\\ & \;-\langle \Psi_{\text{L}}|{Ê}_{t^{m}}^{i^{\prime m}}(Ĥ-i\hat{X})|\Psi_{\text{R}}\rangle +\langle \Psi_{\text{R}}|{Ê}_{t^{m}}^{i^{\prime m}}(Ĥ-i\hat{X})|\Psi_{\text{L}}\rangle =0\;. \end{array}$$

When the CC‐parameterization is equivalent to the CAS, Equation (22) are identities, giving undetermined EOMs of $X_{p^{m}}^{\alpha^{m}}$ and $X_{i^{\prime m}}^{t^{m}}$. In the case of incomplete CC parameterization, one can expect that $ℑ\int_{t_{0}}^{t_{1}}Ldt$ can be close to degenerate in some cases [51], and potential numerical instabilities could happen. Nonetheless, it is interesting to investigate the methods based on the imaginary part of the action when the dynamical cores and virtual orbitals do not exist.

## 3 TD‐oqoCC‐NB Methods

In this section, we will formulate all TD‐oqoCC‐NB methods, and discuss their key properties and the application to vibrational dynamics and electron dynamics.

### 3.1 EOMs of CC Amplitudes and Active Orbital Rotations Based on the Imaginary Action

Before discussing the details of NB methods, we will first derive EOMs of CC amplitudes and active orbital rotations, derived from the imaginary action. The explicit expression of the variation is

(23) $$\begin{array}{c} \begin{array}{ll} 2i\delta S_{I} & =\delta \tau_{\overset{˚}{I}}^{\overset{˚}{A}}\left\{\langle \Psi_{\text{L}}|[Ĥ-i\hat{X},{Ê}_{\overset{˚}{I}}^{\overset{˚}{A}}]|\Psi_{\text{R}}\rangle +i{\dot{\lambda }}_{\overset{˚}{A}}^{\overset{˚}{I}}\right\}\\ & \;+\delta \lambda_{\overset{˚}{A}}^{\overset{˚}{I}}\left\{\langle \Phi_{\overset{˚}{I}}^{\overset{˚}{A}}|e^{-\hat{T}}(Ĥ-i\hat{X})|\Psi_{\text{R}}\rangle -i{\dot{\tau }}_{\overset{˚}{I}}^{\overset{˚}{A}}\right\}-c.c\\ & \;+\underset{m}{\sum }\Delta_{\nu^{m}}^{\mu^{m}}\left\{\left\langle \Psi_{\text{L}}|[Ĥ-i(\partial_{t}^{\prime }\hat{T})-i\hat{X},{Ê}_{\nu^{m}}^{\mu^{m}}]|\Psi_{\text{R}}\right\rangle \right.\\ & \;+i\langle \Phi |(\partial_{t}^{\prime }\hat{\Lambda })e^{-\hat{T}}{Ê}_{\nu^{m}}^{\mu^{m}}|\Psi_{\text{R}}\rangle \\ & \;+{\left\langle \Psi_{\text{L}}|[Ĥ-i(\partial_{t}^{\prime }\hat{T})-i\hat{X},{Ê}_{\mu^{m}}^{\nu^{m}}]|\Psi_{\text{R}}\right\rangle }^{*}\\ & \;\left.-i{\left\langle \Phi |(\partial_{t}^{\prime }\hat{\Lambda })e^{-\hat{T}}{Ê}_{\mu^{m}}^{\nu^{m}}|\Psi_{\text{R}}\right\rangle }^{*}\right\}\;.\; \end{array} \end{array}$$

Clearly, the variation with respect to CC amplitudes yields EOMs Equations ((5), (6), (8), (9)), identical to those in the real action case. The variation of $\delta S_{I}$ with respect to $\Delta_{a^{m}}^{i^{m}}$ gives

(24) $$\begin{array}{cc} & \;\langle \Psi_{\text{L}}|[Ĥ-i\hat{X}-i(\partial_{t}^{\prime }\hat{T}),{Ê}_{a^{m}}^{i^{m}}]|\Psi_{\text{R}}\rangle \\ + & i\langle \Phi |(\partial_{t}^{\prime }\hat{\Lambda })e^{-\hat{T}}{Ê}_{a^{m}}^{i^{m}}|\Psi_{\text{R}}\rangle \\ + & {\left\langle \Psi_{\text{L}}|[Ĥ-i\hat{X}-i(\partial_{t}^{\prime }\hat{T}),{Ê}_{i^{m}}^{a^{m}}]|\Psi_{\text{R}}\right\rangle }^{*}\\ - & i{\left\langle \Phi |(\partial_{t}^{\prime }\hat{\Lambda })e^{-\hat{T}}{Ê}_{i^{m}}^{a^{m}}|\Psi_{\text{R}}\right\rangle }^{*}=0\;, \end{array}$$

Similarly to a real action scenario [65], we get

(25) $$\begin{array}{cc} & \frac{i}{2}(\delta_{b^{m}}^{a^{m}}\rho_{i^{m}}^{j^{m}}-\rho_{b^{m}}^{a^{m}}\delta_{i^{m}}^{j^{m}}){\dot{\tau }}_{j^{m}}^{b^{m}}\\ & \;+i(\delta_{b^{m}}^{a^{m}}K_{i^{m}}^{j^{m}}-K_{b^{m}}^{a^{m}}\delta_{i^{m}}^{j^{m}})X_{j^{m}}^{b^{m}}-i\frac{1}{2}{\left({\dot{\lambda }}_{a^{m}}^{i^{m}}\right)}^{*}=\\ & {\tilde{B}}_{i^{m}}^{a^{m}}-i\frac{1}{2}\underset{n}{\sum }\left\{\langle \Phi_{j^{n}}^{b^{n}}|e^{-\hat{T}}{Ê}_{a^{m}}^{i^{m}}|\Psi_{\text{R}}\rangle {\dot{\lambda }}_{b^{n}}^{j^{n}}+A_{i^{m}b^{n}}^{a^{m}j^{n}}{\left(X_{j^{n}}^{b^{n}}\right)}^{*}\right\}\;, \end{array}$$

where

(26) $$\begin{array}{c} \begin{array}{ll} {\tilde{B}}_{i^{m}}^{a^{m}} & =-\frac{1}{2}\langle \Psi_{\text{L}}|[Ĥ,{Ê}_{a^{m}}^{i^{m}}]|\Psi_{\text{R}}\rangle +\frac{1}{2}\langle \Psi_{\text{R}}|[Ĥ,{Ê}_{a^{m}}^{i^{m}}]|\Psi_{\text{L}}\rangle \\ & \;-\frac{1}{2}\langle \Psi_{\text{L}}|[{Ê}_{I}^{A},Ĥ]|\Psi_{\text{R}}\rangle \langle \Phi_{I}^{A}|e^{-\hat{T}}{Ê}_{a^{m}}^{i^{m}}|\Psi_{\text{R}}\rangle \\ & \;+\frac{1}{2}\langle \Psi_{\text{L}}|[{Ê}_{I}^{A},{Ê}_{a^{m}}^{i^{m}}]|\Psi_{\text{R}}\rangle \langle \Phi_{I}^{A}|e^{-\hat{T}}Ĥ|\Psi_{\text{R}}\rangle \;,\; \end{array} \end{array}$$

(27) $$\begin{array}{ll} K_{q^{m}}^{p^{m}} & =\frac{1}{2}(\rho_{q^{m}}^{p^{m}}-{\left(\rho_{p^{m}}^{q^{m}}\right)}^{*})\;.\; \end{array}$$

#### 3.1.1 TD‐OCC Analog

Using the same parameterization as the TD‐OCC, we have

(28) $$i(\delta_{b^{m}}^{a^{m}}K_{i^{m}}^{j^{m}}-K_{b^{m}}^{a^{m}}\delta_{i^{m}}^{j^{m}})X_{j^{m}}^{b^{m}}+i\underset{n}{\sum }\frac{1}{2}A_{i^{m}b^{n}}^{a^{m}j^{n}}{\left(X_{j^{n}}^{b^{n}}\right)}^{*}={\tilde{B}}_{i^{m}}^{a^{m}},$$

for $\delta S_{I}$.

It is important to emphasize that TD‐OCC analog could be potentially unstable, even ill‐defined. Considering the case of a two‐electron system, the ansatz can represent the FCI wave function faithfully, leading to $K_{q^{m}}^{p^{m}}={\tilde{B}}_{i^{m}}^{a^{m}}=0$ and $A_{i^{m}b^{n}}^{a^{m}j^{n}}=0$. Therefore, Equation (28) becomes meaningless. This is similar to the EOMs of $X_{p^{m}}^{\alpha^{m}}$ and $X_{i^{\prime m}}^{t^{m}}$ from $\delta S_{I}=0$, corresponding to the “diagonal” manifold. We can also expect that the manifold may be close to the degenerate, leading to instability if the parameterization is good enough to approximate TD‐CASSCF or FCI.

#### 3.1.2 TD‐BCC Analog

Using the same parameterization as the TD‐BCC, we have Equation (18) and

(29) $$\begin{array}{ll} & i\frac{1}{2}\left\{\underset{n}{\sum }\langle \Phi_{j^{n}}^{b^{n}}|e^{-\hat{T}}{Ê}_{a^{m}}^{i^{m}}|\Psi_{\text{R}}\rangle {\dot{\lambda }}_{b^{n}}^{j^{n}}-{\left({\dot{\lambda }}_{a^{m}}^{i^{m}}\right)}^{*}\right\}=\\ & {\tilde{B}}_{i^{m}}^{a^{m}}-i(\delta_{b^{m}}^{a^{m}}K_{i^{m}}^{j^{m}}-K_{b^{m}}^{a^{m}}\delta_{i^{m}}^{j^{m}})X_{j^{m}}^{b^{m}}-i\underset{n}{\sum }\frac{1}{2}A_{i^{m}b^{n}}^{a^{m}j^{n}}{\left(X_{j^{n}}^{b^{n}}\right)}^{*}\;,\; \end{array}$$

for $\delta S_{I}$.

The difference of Equations ((19), (29)) (or Equations ((11), (25))) gives

(30) $$i{\left({\dot{\lambda }}_{a^{m}}^{i^{m}}\right)}^{*}={\left\langle \Psi_{\text{L}}|[Ĥ-i\hat{X},{Ê}_{i^{m}}^{a^{m}}]|\Psi_{\text{R}}\right\rangle }^{*},$$

which coincides with Equation (9). It is known that Equation (19), (9) are equivalent when TD‐BCC converges to FCI or TD‐CASSCF limit; therefore, one can expect that Equation (29) also gives the TD‐CASSCF result, which demonstrates the validity of $\delta S_{I}$.

#### 3.1.3 TD‐OCCT1 and TD‐OCCX0 Analog

Using the same parameterization as the TD‐OCCT1, we have Equation (16) and

(31) $$\begin{array}{cc} & \frac{i}{2}(\delta_{b^{m}}^{a^{m}}\rho_{i^{m}}^{j^{m}}-\rho_{b^{m}}^{a^{m}}\delta_{i^{m}}^{j^{m}}){\dot{\tau }}_{j^{m}}^{b^{m}}=\\ & {\tilde{B}}_{i^{m}}^{a^{m}}-i(\delta_{b^{m}}^{a^{m}}K_{i^{m}}^{j^{m}}-K_{b^{m}}^{a^{m}}\delta_{i^{m}}^{j^{m}})X_{j^{m}}^{b^{m}}-i\underset{n}{\sum }\frac{1}{2}A_{i^{m}b^{n}}^{a^{m}j^{n}}{\left(X_{j^{n}}^{b^{n}}\right)}^{*}\;. \end{array}$$

for $\delta S_{I}$. One can verify that Equation (17) and (31) are completely equivalent due to Equation (16). This result is unsurprising since TD‐OCCT1 is gauge equivalent to TD‐NOCC when virtual orbitals and dynamical cores are absent, which suggests that the active wave function parameterization should be complex analytical.

The TD‐OCCX0 parameterization is also complex analytical, and the imaginary action cannot lead to new results, either.

### 3.2 EOMs of TD‐oqoCC‐NB Methods

The key idea of TD‐oqoCC‐NB methods is to select constraints (from $\tau_{i^{m}}^{a^{m}},\lambda_{a^{m}}^{i^{m}}$, or $X_{i^{m}}^{a^{m}}=0$ in the LG) and EOMs (from Equations ((8), (9), (11), (25))) for CC amplitudes and particle‐hole orbital rotations without necessarily obeying the TD‐BIVP. Here, one can immediately see the major difference between NB methods and TD‐ooCC methods, that is, the selection of EOMs is not solely determined by the ansatz. For this reason, we have to derive all EOMs including all single excitations and deexcitations as well as particle‐hole orbital rotations without constraints [Equations ((8), (9), (11), (25))] first, then set constraints in EOMs. Similar to TD‐ooCC methods, constraints are important for the numerical stability at finite truncations. All NB methods use the constraints $X_{i^{m}}^{j^{m}}=X_{a^{m}}^{b^{m}}=0$ in the LG to make sure the numerical stability, and for some of them $X_{i^{m}}^{j^{m}},X_{a^{m}}^{b^{m}}$ are redundant variables, which will be discussed in Section 3.4.2. The orbital rotations other than active‐active are given by Equation (A1), that is, determined by TD‐BIVP from the real action. As the name suggests, NB methods do not obey TD‐BIVP in general, and orbital rotations can be considered as quasi‐optimal. There are plenty of possible TD‐oqoCC‐NB, we will list all of them here. They are named as TD‐oqoCC‐NBm, where m is the index of the list. The main results of NB methods, including the constraints, selected EOMs, and important properties are listed in Table 2. We also state the constraints and selected EOMs here explicitly for completeness.

**TABLE 2:** Characteristics of TD‐oqoCC‐NB methods and TD‐ooCC methods.

Method	Constraints	Selected	Converge to	ph gauge	pp & hh rotation	Gauge	Ehrenfest	No vibrational
	(≡0)	EOMs	TD‐CASSCF	Invariance	(gauge) invariance	Invariance	Theorem^a^	Inverse
NB1	$\tau_{i^{m}}^{a^{m}},\lambda_{a^{m}}^{i^{m}},\delta \tau_{i^{m}}^{a^{m}},\delta \lambda_{a^{m}}^{i^{m}}$	Equation (8)	No	Yes	Yes	Yes	No	No
NB2		Equation (9)	No	Yes	Yes	Yes	No	Yes
NB3^b^, ^c^		Equation (25)	No	Yes	Yes	Yes	No	No^d^
TD‐OCC		Equation (11)	No	Yes	Yes	Yes	Yes	No^d^
NB4	$\tau_{i^{m}}^{a^{m}},\delta \tau_{i^{m}}^{a^{m}},X_{i^{m}}^{a^{m}},\Delta_{i^{m}}^{a^{m}}$	Equation (9)	No	No	No	No	No	Yes
NB5		Equation (11)	No	No	No	No	No	No
NB6		Equation (25)	No	No	No	No	No	No
NB7	$\lambda_{a^{m}}^{i^{m}},\delta \lambda_{a^{m}}^{i^{m}},X_{i^{m}}^{a^{m}},\Delta_{i^{m}}^{a^{m}}$	Equation (8)	No	No	No	No	No	Yes
NB8		Equation (11)	No	No	No	No	No	Yes
NB9		Equation (25)	No	No	No	No	No	Yes
NB10	$\lambda_{a^{m}}^{i^{m}},\delta \lambda_{a^{m}}^{i^{m}}$	Equations ((8), (9))	Yes	Yes	Yes	Yes	No	Yes
NB11		Equations ((8), (11))	Yes	Yes	No	No	No	No
NB12		Equations ((8), (25))	Yes	Yes	No	No	No	No
TD‐OCCT1		Equations ((9), (11))	Yes	Yes	Yes	Yes	Yes	Yes
NB13	$\tau_{i^{m}}^{a^{m}},\delta \tau_{i^{m}}^{a^{m}}$	Equations ((8), (9))	Yes	Yes	Yes	Yes	No	No
NB14		Equations ((9), (11))	Yes	Yes	No	No	No	No
NB15^b^		Equations ((8), (25))	Yes	Yes	Yes	Yes	No	No
TD‐BCC		Equations ((8), (11))	Yes	Yes	Yes	Yes	Yes	No
NB16	$X_{i^{m}}^{a^{m}},\Delta_{i^{m}}^{a^{m}}$	Equations ((8), (10))	Yes	No	No	No	No	No
NB17		Equations (8) and (25)	Yes	No	No	No	No	No
NB18		Equations (9) and (11)	Yes	No	No	No	No	Yes
TD‐OCCX0		Equation (8) and (9)	Yes	No	Yes	No	No	Yes

^a^ Witout frozen‐core orbitals.

^b^ Imaginary action if without virtual orbitals and dynamical cores.

^c^ Potentially unstable.

^d^ Yes if only even excitations are included.

For NB1 to NB3, under the condition $\tau_{i^{m}}^{a^{m}}=\lambda_{a^{m}}^{i^{m}}=\delta \tau_{i^{m}}^{a^{m}}=\delta \lambda_{a^{m}}^{i^{m}}\equiv 0$, different EOMs are selected as follows: (1): Equation (8) is selected; (2): Equation (9) is selected; (3): Equation (25) is selected.

For NB4 to NB6, under the condition $\tau_{i^{m}}^{a^{m}}=\delta \tau_{i^{m}}^{a^{m}}=X_{i^{m}}^{a^{m}}=\Delta_{i^{m}}^{a^{m}}\equiv 0$, different EOMs are selected as follows: (4): Equation (9) is selected; (5): Equation (11) is selected; (6): Equation (25) is selected.

For NB7 to NB9, under the condition $\lambda_{a^{m}}^{i^{m}}=\delta \lambda_{a^{m}}^{i^{m}}=X_{i^{m}}^{a^{m}}=\Delta_{i^{m}}^{a^{m}}\equiv 0$, different EOMs are selected as follows: (7): Equation (8) is selected; (8): Equation (11) is selected; (9): Equation (25) is selected.

For NB10 to NB12, under the condition $\lambda_{a^{m}}^{i^{m}}=\delta \lambda_{a^{m}}^{i^{m}}\equiv 0$, different EOMs are selected as follows: (10): Equations (8) and (9) are selected; (11): Equations (8) and (11) are selected; (12): Equations (8) and (25) are selected.

For NB13 to NB15, under the condition $\tau_{i^{m}}^{a^{m}}=\delta \tau_{i^{m}}^{a^{m}}\equiv 0$, different EOMs are selected as follows: (13): Equations ((8), (9)) are selected; (14): Equations ((9), (11)) are selected; (15): Equations ((8), (25)) are selected.

For NB16 to NB18, under the condition $X_{i^{m}}^{a^{m}}=\Delta_{i^{m}}^{a^{m}}\equiv 0$, different EOMs are selected as follows: (16): Equations ((8), (11)) are selected; (17): Equations ((8), (25)) are selected; (18): Equations ((9), (11)) are selected.

NB10 to NB18 can converge to the TD‐CASSCF. For methods where two equations are selected, there are only four non‐duplicated combinations (using in NB methods): Equations (9), (25), (11), and (25) are equivalent to Equations (9) and (11). We also excluded variational methods and inconsistent combinations. More explicitly, $\tau_{i^{m}}^{a^{m}}=\delta \tau_{i^{m}}^{a^{m}}=X_{i^{m}}^{a^{m}}=\Delta_{i^{m}}^{a^{m}}\equiv 0$ with Equation (8) and $\lambda_{a^{m}}^{i^{m}}=\delta \lambda_{a^{m}}^{i^{m}}=X_{i^{m}}^{a^{m}}=\Delta_{i^{m}}^{a^{m}}\equiv 0$ with Equation (9) are inconsistent; $\tau_{i^{m}}^{a^{m}}=\lambda_{a^{m}}^{i^{m}}=\delta \tau_{i^{m}}^{a^{m}}=\delta \lambda_{a^{m}}^{i^{m}}\equiv 0$ with Equation (11) is TD‐OCC, $\lambda_{a^{m}}^{i^{m}}=\delta \lambda_{a^{m}}^{i^{m}}\equiv 0$ with Equations (9) and (11) is TD‐OCCT1, $\tau_{i^{m}}^{a^{m}}=\delta \tau_{i^{m}}^{a^{m}}\equiv 0$ with Equations (8) and (11) is TD‐BCC, and $X_{i^{m}}^{a^{m}}=\Delta_{i^{m}}^{a^{m}}\equiv 0$ with Equations (8) and (9) is TD‐OCCX0. Additionally, NB3 and NB15 are TD‐OCC and TD‐BCC analogs with imaginary action when virtual orbitals and dynamical cores do not exist.

Defining superoperators as in Appendix A.2, EOMs of NB methods and TD‐ooCC can be re‐expressed in compact forms, listed in Table 3. More explicit EOMs of NB methods are available in Appendix A.3.

**TABLE 3:** Mathematical structure and block‐matrix working equations of the 18 NB methods and reference methods.

Method	Variables (solution order)	Unified working equation form (i𝕊·v=F)
NB1	X	$i{𝕀}_{O}[X]=R_{0}$
NB2	X	$i{𝕄}_{\rho^{T}}[X]=L_{0}$
NB3	X	$i{𝕄}_{K}[X]+i\frac{1}{2}{𝒪}_{2}(X^{*})=\tilde{B}$
NB4	$\dot{\lambda }$	$-i𝕀[\dot{\lambda }]=L_{X}$
NB5	$\dot{\lambda }$	$i{\tilde{𝕃}}_{+}[\dot{\lambda }]=B_{D}[X]$
NB6	$\dot{\lambda }$	$i{\tilde{𝕃}}_{-}[\dot{\lambda }]=B_{K}[X]$
NB7	$\dot{\tau }$	$i𝕀[\dot{\tau }]=R_{X}$
NB8	$\dot{\tau }$	$i\frac{1}{2}{𝕄}_{\rho }[\dot{\tau }]=B_{D}[X]$
NB9	$\dot{\tau }$	$i\frac{1}{2}{𝕄}_{\rho }[\dot{\tau }]=B_{K}[X]$
NB10	$X\to \dot{\tau }$	$\left\{i{𝕄}_{\rho^{T}}[X]=L_{0}\}\;\to \;\{i𝕀[\dot{\tau }]=R_{X}\right\}$
NB11	$X\to \dot{\tau }$	$\left\{i{𝕎}_{+}[X]=B-\frac{1}{2}{𝕄}_{\rho }[R_{0}]\}\;\to \;\{i𝕀[\dot{\tau }]=R_{X}\right\}$
NB12	$X\to \dot{\tau }$	$\left\{i{𝕎}_{-}[X]=\tilde{B}-\frac{1}{2}{𝕄}_{\rho }[R_{0}]\}\;\to \;\{i𝕀[\dot{\tau }]=R_{X}\right\}$
NB13	$X\to \dot{\lambda }$	$\left\{i{𝕀}_{O}[X]=R_{0}\}\;\to \;\{-i𝕀[\dot{\lambda }]=L_{X}\right\}$
NB14	$X\to \dot{\lambda }$	$\left\{i𝕌[X]=B+\frac{1}{2}{\tilde{𝒪}}_{1}(L_{0})-\frac{1}{2}L_{0}^{*}\}\;\to \;\{-i𝕀[\dot{\lambda }]=L_{X}\right\}$
NB15	$X\to \dot{\lambda }$	$\left\{i{𝕀}_{O}[X]=R_{0}\}\;\to \;\{i{\tilde{𝕃}}_{-}[\dot{\lambda }]=B_{K}[X]\right\}$
NB16	$\dot{\tau }\to \dot{\lambda }$	$i\left(\begin{array}{cc} 𝕀 & 0\\ \frac{1}{2}{𝕄}_{\rho } & {\tilde{𝕃}}_{+} \end{array}\right)\left(\begin{array}{c} \dot{\tau }\\ \dot{\lambda } \end{array}\right)=\left(\begin{array}{c} R_{X}\\ B_{D}[X] \end{array}\right)$
NB17	$\dot{\tau }\to \dot{\lambda }$	$i\left(\begin{array}{cc} 𝕀 & 0\\ \frac{1}{2}{𝕄}_{\rho } & {\tilde{𝕃}}_{-} \end{array}\right)\left(\begin{array}{c} \dot{\tau }\\ \dot{\lambda } \end{array}\right)=\left(\begin{array}{c} R_{X}\\ B_{K}[X] \end{array}\right)$
NB18	$\dot{\lambda }\to \dot{\tau }$	$i\left(\begin{array}{cc} \frac{1}{2}{𝕄}_{\rho } & {\tilde{𝕃}}_{+}\\ 0 & -𝕀 \end{array}\right)\left(\begin{array}{c} \dot{\tau }\\ \dot{\lambda } \end{array}\right)=\left(\begin{array}{c} B_{D}[X]\\ L_{X} \end{array}\right)$
TD‐OCC	X	$B_{D}[X]=0\;(\text{or}\;i{𝕄}_{D}[X]+i\frac{1}{2}{𝒪}_{2}(X^{*})=B)$
TD‐OCCT1	$X\to \dot{\tau }$	$\left\{i{𝕄}_{\rho^{T}}[X]=L_{0}\}\;\to \;\{i\frac{1}{2}{𝕄}_{\rho }[\dot{\tau }]=B_{D}[X]\right\}$
TD‐BCC	$X\to \dot{\lambda }$	$\left\{i{𝕀}_{O}[X]=R_{0}\}\;\to \;\{i{\tilde{𝕃}}_{+}[\dot{\lambda }]=B_{D}[X]\right\}$
TD‐OCCX0	$\dot{\tau },\dot{\lambda }$	$\left\{i𝕀[\dot{\tau }]=R_{X}\}\;\text{and}\;\{-i𝕀[\dot{\lambda }]=L_{X}\right\}$
Overparam. (real action)	$\dot{\tau },\dot{\lambda },X$	$\left\{i𝕀[\dot{\tau }]=R_{X},\;-i𝕀[\dot{\lambda }]=L_{X},B_{D}[X]=i\frac{1}{2}{𝕄}_{\rho }[\dot{\tau }]+i{\tilde{𝕃}}_{+}[\dot{\lambda }]\right\}$
Overparam. (imaginary action)	$\dot{\tau },\dot{\lambda },X$	$\left\{i𝕀[\dot{\tau }]=R_{X},\;-i𝕀[\dot{\lambda }]=L_{X},B_{K}[X]=i\frac{1}{2}{𝕄}_{\rho }[\dot{\tau }]+i{\tilde{𝕃}}_{-}[\dot{\lambda }]\right\}$

In what follows, we will discuss important properties of NB methods for vibrational and electron dynamics, including the inverse matrix operations in EOMs, gauge and rotation invariance, and the Ehrenfest theorem. The results are summarized in Table 2. As we demonstrated in the introduction, a valuable list tailored to each scenario guiding future numerical benchmarking and important modifications on observable evaluations are provided.

### 3.3 Systems With Large Number of Fermion Modes

It is numerically unfavorable if the inverse of a modes‐coupling matrix appears in EOMs because of the huge matrix dimension if many fermion modes exist. NB2, NB4, NB7 to 10, and NB18 avoid inverse of matrix composed of different modes. For comparison, in the TD‐ooCC family, TD‐OCCT1 and TD‐OCCX0 satisfy this property, while TD‐OCC and TD‐BCC do not. In addition, the NB3 method does not contain the inverse of matrix composed of different modes if only even excitations are included in the ansatz, which is similar to the TD‐OCC method. One of the typical systems is vibrational dynamics (isomorphic to single‐occupation in each fermion kind). Vibronic coupling systems and non‐adiabatic dynamics also belong to this case (both in the traditional framework [66, 67, 68] and in the equal footing treatment on electronic and nuclear DOF [26, 69, 70, 71, 72, 73].) Another example is the periodic systems, in which different k‐mode fermions can be formally considered as different fermion modes in EOMs of single CC amplitudes and orbital rotations.

### 3.4 Gauge Invariance

Gauge invariance is crucial for the electron dynamics simulation. For methods violating the gauge invariance, the orbital rotation constraints (set to be zero) have to be understood in the LG, and the VG constraints are adjusted to hold the gauge invariance [47]. Details will be discussed in Section 3.6. In this subsection, we will focus on the gauge invariance examination of NB methods. The TD‐ooCC family is gauge invariant (if there is no frozen‐core orbitals) except for the TD‐OCCX0.

#### 3.4.1 particle‐Hole Gauge Invariance

As long as $X_{i^{m}}^{a^{m}}$ are variables, all methods considered in this article are particle‐hole gauge‐invariant since Ĥ and $\hat{X}$ always appear as a combination $Ĥ-i\hat{X}$ in all EOMs. The list is: NB1 to NB3, NB10 to NB15.

#### 3.4.2 Particle‐Particle and Hole‐Hole Rotation (Gauge) Invariance

The hh and pp rotation invariance holds if $\delta S_{R}/\delta \Delta_{i^{m}}^{j^{m}}=\delta S_{R}/\delta \Delta_{a^{m}}^{b^{m}}=0$. In this case, $X_{i^{m}}^{j^{m}},X_{a^{m}}^{b^{m}}$ are redundant variables. We select non‐single excitations as Equation (7), and we will show that the condition does not ensure the rotation invariance for NB methods in general.

Following the derivation in Reference [26],

(32) $$\begin{array}{c} \begin{array}{ll} & \;\delta \int_{t_{0}}^{t_{1}}Ldt=\langle \Psi_{\text{L}}|[Ĥ-i(\partial_{t}^{\prime }\hat{T})-i\hat{X},{Ê}_{\nu^{m}}^{\mu^{m}}]|\Psi_{\text{R}}\rangle \\ + & i\langle \Phi |(\partial_{t}^{\prime }\hat{\Lambda })e^{-\hat{T}}{Ê}_{\nu^{m}}^{\mu^{m}}|\Psi_{\text{R}}\rangle \\ = & \left\langle \Psi_{\text{L}}|[Ĥ-i\hat{X},{Ê}_{\nu^{m}}^{\mu^{m}}]|\Psi_{\text{R}}\right\rangle \\ - & i{\dot{\tau }}_{\overset{˚}{I}}^{\overset{˚}{A}}\langle \Psi_{\text{L}}|[{Ê}_{\overset{˚}{I}}^{\overset{˚}{A}},{Ê}_{\nu^{m}}^{\mu^{m}}]|\Psi_{\text{R}}\rangle +i{\dot{\lambda }}_{\overset{˚}{A}}^{\overset{˚}{I}}\langle \Phi_{\overset{˚}{I}}^{\overset{˚}{A}}|e^{-\hat{T}}{Ê}_{\nu^{m}}^{\mu^{m}}|\Psi_{\text{R}}\rangle \; \end{array} \end{array}$$

(33) $$\begin{array}{c} \begin{array}{ll} = & \left\langle \Psi_{\text{L}}|{Ê}_{\overset{˚}{I}}^{\overset{˚}{A}}(Ĥ-i\hat{X})|\Psi_{\text{R}}\rangle \langle \Phi_{\overset{˚}{I}}^{\overset{˚}{A}}|e^{-\hat{T}}{Ê}_{\nu^{m}}^{\mu^{m}}|\Psi_{\text{R}}\right\rangle \\ - & \left\langle \Phi_{\overset{˚}{I}}^{\overset{˚}{A}}|e^{-\hat{T}}(Ĥ-i\hat{X})|\Psi_{\text{R}}\rangle \langle \Psi_{\text{L}}|{Ê}_{\overset{˚}{I}}^{\overset{˚}{A}}{Ê}_{\nu^{m}}^{\mu^{m}}|\Psi_{\text{R}}\right\rangle \\ - & \underset{m}{\sum }(i{\dot{\tau }}_{i^{m}}^{a^{m}}-\langle \Phi_{i^{m}}^{a^{m}}|e^{-\hat{T}}(Ĥ-i\hat{X})|\Psi_{\text{R}}\rangle )\langle \Psi_{\text{L}}|[{Ê}_{i^{m}}^{a^{m}},{Ê}_{\nu^{m}}^{\mu^{m}}]|\Psi_{\text{R}}\rangle \\ + & \underset{m}{\sum }(i{\dot{\lambda }}_{a^{m}}^{i^{m}}+\langle \Psi_{\text{L}}|[Ĥ-i\hat{X},{Ê}_{i^{m}}^{a^{m}}]|\Psi_{\text{R}}\rangle )\langle \Phi_{i^{m}}^{a^{m}}|e^{-\hat{T}}{Ê}_{\nu^{m}}^{\mu^{m}}|\Psi_{\text{R}}\rangle \; \end{array} \end{array}$$

(34) $$\begin{array}{ll} = & \underset{m}{\sum }(i{\dot{\lambda }}_{a^{m}}^{i^{m}}+\langle \Psi_{\text{L}}|[Ĥ-i\hat{X},{Ê}_{i^{m}}^{a^{m}}]|\Psi_{\text{R}}\rangle )\langle \Phi_{i^{m}}^{a^{m}}|e^{-\hat{T}}{Ê}_{\nu^{m}}^{\mu^{m}}|\Psi_{\text{R}}\rangle \\ - & \underset{m}{\sum }(i{\dot{\tau }}_{i^{m}}^{a^{m}}-\langle \Phi_{i^{m}}^{a^{m}}|e^{-\hat{T}}(Ĥ-i\hat{X})|\Psi_{\text{R}}\rangle )\langle \Psi_{\text{L}}|[{Ê}_{i^{m}}^{a^{m}},{Ê}_{\nu^{m}}^{\mu^{m}}]|\Psi_{\text{R}}\rangle \;.\; \end{array}$$

Therefore, there are four scenarios that $\delta S_{R}/\delta \Delta_{i^{m}}^{j^{m}}=\delta S_{R}/\delta \Delta_{a^{m}}^{b^{m}}=0$ can be satisfied: (1) both $\left\langle \Psi_{\text{L}}|[{Ê}_{i^{m}}^{a^{m}},{Ê}_{\nu^{m}}^{\mu^{m}}]|\Psi_{\text{R}}\right\rangle$ (corresponding to no ${\hat{\Lambda }}_{1}$ in the ansatz) and $\left\langle \Phi_{i^{m}}^{a^{m}}|e^{-\hat{T}}{Ê}_{\nu^{m}}^{\mu^{m}}|\Psi_{\text{R}}\right\rangle$ (corresponding to no ${\hat{T}}_{1}$ in the ansatz) are zero, including NB1 to 3 (and TD‐OCC); (2) $\left\langle \Psi_{\text{L}}|[{Ê}_{i^{m}}^{a^{m}},{Ê}_{\nu^{m}}^{\mu^{m}}]|\Psi_{\text{R}}\right\rangle$ and $\left(i{\dot{\lambda }}_{a^{m}}^{i^{m}}+\langle \Psi_{\text{L}}|[Ĥ-i\hat{X},{Ê}_{i^{m}}^{a^{m}}]|\Psi_{\text{R}}\rangle \right)$ are zero, including NB10 (and TD‐OCCT1); (3) $\left\langle \Phi_{i^{m}}^{a^{m}}|e^{-\hat{T}}{Ê}_{\nu^{m}}^{\mu^{m}}|\Psi_{\text{R}}\right\rangle$ and $\left(i{\dot{\tau }}_{i^{m}}^{a^{m}}-\langle \Phi_{i^{m}}^{a^{m}}|e^{-\hat{T}}(Ĥ-i\hat{X})|\Psi_{\text{R}}\rangle \right)$ are zero, including NB13, NB15 (and TD‐BCC); (4) $\left(i{\dot{\tau }}_{i^{m}}^{a^{m}}-\langle \Phi_{i^{m}}^{a^{m}}|e^{-\hat{T}}(Ĥ-i\hat{X})|\Psi_{\text{R}}\rangle \right)$ and $\left(i{\dot{\lambda }}_{a^{m}}^{i^{m}}+\langle \Psi_{\text{L}}|[Ĥ-i\hat{X},{Ê}_{i^{m}}^{a^{m}}]|\Psi_{\text{R}}\rangle \right)$ are zero, including no NB methods (only TD‐OCCX0 belongs to this scenario).

In summary, NB1 to 3, NB10, Nb13, and NB15 are gauge invariant.

### 3.5 Ehrenfest Theorem

The gauge invariance of the TD‐ooCC family can ensure the Ehrenfest theorem, however, it is not the case for NB methods. In fact, none of NB methods can obey the Ehrenfest theorem, and one has to use the modified one for the calculation of the time derivative of an operator expectation value, e.g., velocity and acceleration as time derivative(s) of the position, which is important for simulating high harmonic generation (HHG) process in strong field‐induced electron dynamics [47].

It is straightforward to verify that

(35) $$\begin{array}{ll} & i\frac{d}{dt}|\Psi_{\text{R}}\rangle =i\hat{X}|\Psi_{\text{R}}\rangle +\underset{m}{\sum }e^{\hat{T}}|\Phi_{j^{m}}^{b^{m}}\rangle (i{\dot{\tau }}_{j^{m}}^{b^{m}}-\langle \Phi_{j^{m}}^{b^{m}}|e^{-\hat{T}}(Ĥ-i\hat{X})|\Psi_{\text{R}}\rangle )\\ & \;+e^{\hat{T}}\hat{\Pi }e^{-\hat{T}}(Ĥ-i\hat{X})|\Psi_{\text{R}}\rangle \;,\; \end{array}$$

(36) $$\begin{array}{c} \begin{array}{ll} & i\frac{d}{dt}\langle \Psi_{\text{L}}|=-i\langle \Psi_{\text{L}}|\hat{X}\\ & \;+\underset{m}{\sum }(\langle \Phi_{j^{m}}^{b^{m}}|e^{-\hat{T}}(Ĥ-i\hat{X})|\Psi_{\text{R}}\rangle -i{\dot{\tau }}_{j^{m}}^{b^{m}})\langle \Psi_{\text{L}}|{Ê}_{j^{m}}^{b^{m}}\\ & \;+\underset{m}{\sum }(i{\dot{\lambda }}_{b^{m}}^{j^{m}}+\langle \Psi_{\text{L}}|[Ĥ-i\hat{X},{Ê}_{j^{m}}^{b^{m}}]|\Psi_{\text{R}}\rangle )\langle \Phi_{j^{m}}^{b^{m}}|e^{-\hat{T}}\\ & \;-\langle \Psi_{\text{L}}|(Ĥ-i\hat{X})e^{\hat{T}}\hat{\Pi }e^{-\hat{T}}\;,\; \end{array} \end{array}$$

(37) $$\begin{array}{c} \begin{array}{ll} & i\frac{d}{dt}\langle \Psi_{\text{L}}|Ô|\Psi_{\text{R}}\rangle -\langle \Psi_{\text{L}}|[Ô,Ĥ]|\Psi_{\text{R}}\rangle =\langle \Psi_{\text{L}}|(Ĥ-i\hat{X})e^{\hat{T}}\bar{\hat{\Pi }}e^{-\hat{T}}Ô|\Psi_{\text{R}}\rangle \\ & \;-\langle \Psi_{\text{L}}|Ôe^{\hat{T}}\bar{\hat{\Pi }}e^{-\hat{T}}(Ĥ-i\hat{X})|\Psi_{\text{R}}\rangle \\ & \;+\underset{m}{\sum }(i{\dot{\lambda }}_{b^{m}}^{j^{m}}+\langle \Psi_{\text{L}}|[Ĥ-i\hat{X},{Ê}_{j^{m}}^{b^{m}}]|\Psi_{\text{R}}\rangle )\langle \Phi_{j^{m}}^{b^{m}}|e^{-\hat{T}}Ô|\Psi_{\text{R}}\rangle \\ & \;+\underset{m}{\sum }(\langle \Phi_{j^{m}}^{b^{m}}|e^{-\hat{T}}(Ĥ-i\hat{X})|\Psi_{\text{R}}\rangle -i{\dot{\tau }}_{j^{m}}^{b^{m}})\langle \Psi_{\text{L}}|[{Ê}_{j^{m}}^{b^{m}},Ô]|\Psi_{\text{R}}\rangle \;,\; \end{array} \end{array}$$

where Ô is an arbitrary one‐body operator and $\bar{\hat{\Pi }}=Î-\hat{\Pi }$.

TD‐ooCC‐NB and TD‐ooCC can only give violation of the Ehrenfest theorem due to frozen‐core‐non‐virtual rotations ($O_{i^{\prime \prime m}}^{\mu^{m}}$ terms) and active‐active rotations($O_{u^{m}}^{t^{m}}$ terms.) After some simplifications, one has

(38) $$\begin{array}{c} \begin{array}{cc} & 2i\frac{d}{dt}\langle Ô\rangle =i\frac{d}{dt}\langle \Psi_{\text{L}}|Ô|\Psi_{\text{R}}\rangle +i\frac{d}{dt}\langle \Psi_{\text{R}}|Ô|\Psi_{\text{L}}\rangle \\ & \;=\langle \Psi_{\text{L}}|[Ô,Ĥ]|\Psi_{\text{R}}\rangle +\langle \Psi_{\text{R}}|[Ô,Ĥ]|\Psi_{\text{L}}\rangle \\ & \;+\underset{m}{\sum }\left\{O_{a^{m}}^{i^{m}}\left(\left\langle \Psi_{\text{L}}|(Ĥ-i\hat{X})e^{\hat{T}}\bar{\hat{\Pi }}e^{-\hat{T}}{Ê}_{a^{m}}^{i^{m}}|\Psi_{\text{R}}\right\rangle \right.\right.\\ & \;\left.\left.-\langle \Psi_{\text{L}}|{Ê}_{a^{m}}^{i^{m}}e^{\hat{T}}\bar{\hat{\Pi }}e^{-\hat{T}}(Ĥ-i\hat{X})|\Psi_{\text{R}}\rangle \right)-c.c.\right\}\\ & \;+\underset{m}{\sum }\left\{O_{a^{m}}^{i^{m}}\left(i{\dot{\lambda }}_{b^{m}}^{j^{m}}+\langle \Psi_{\text{L}}|[Ĥ-i\hat{X},{Ê}_{j^{m}}^{b^{m}}]|\Psi_{\text{R}}\rangle \right)\right.\\ & \;\left.\times \langle \Phi_{j^{m}}^{b^{m}}|e^{-\hat{T}}{Ê}_{a^{m}}^{i^{m}}|\Psi_{\text{R}}\rangle -c.c.\right\}\\ & \;+\underset{m}{\sum }\left\{O_{a^{m}}^{i^{m}}\left(\langle \Phi_{j^{m}}^{b^{m}}|e^{-\hat{T}}(Ĥ-i\hat{X})|\Psi_{\text{R}}\rangle -i{\dot{\tau }}_{j^{m}}^{b^{m}}\right)\right.\\ & \;\left.\times \langle \Psi_{\text{L}}|[{Ê}_{j^{m}}^{b^{m}},{Ê}_{a^{m}}^{i^{m}}]|\Psi_{\text{R}}\rangle -c.c.\right\}\\ & \;-\underset{m}{\sum }\left\{O_{a^{m}}^{i^{m}}\left({\left\langle \Psi_{\text{L}}|[Ĥ-i\hat{X},{Ê}_{i^{m}}^{a^{m}}]|\Psi_{\text{R}}\right\rangle }^{*}-i{\left({\dot{\lambda }}_{a^{m}}^{i^{m}}\right)}^{*}\right)-c.c.\right\}\\ & \;-\underset{m}{\sum }O_{\mu^{m}}^{i^{\prime \prime m}}\left\{{\left\langle \Psi_{\text{L}}|(Ĥ-i\hat{X}){Ê}_{i^{\prime \prime m}}^{\mu^{m}}|\Psi_{\text{R}}\right\rangle }^{*}\right.\\ & \;\left.+\langle \Psi_{\text{L}}|{Ê}_{\mu^{m}}^{i^{\prime \prime m}}(Ĥ-i\hat{X})|\Psi_{\text{R}}\rangle \right\}\\ & \;+\underset{m}{\sum }O_{i^{\prime \prime m}}^{\mu^{m}}\left\{\left\langle \Psi_{\text{L}}|(Ĥ-i\hat{X}){Ê}_{i^{\prime \prime m}}^{\mu^{m}}|\Psi_{\text{R}}\right\rangle \right.\\ & \;\left.+{\left\langle \Psi_{\text{L}}|{Ê}_{\mu^{m}}^{i^{\prime \prime m}}(Ĥ-i\hat{X})|\Psi_{\text{R}}\right\rangle }^{*}\right\}\\ & \;+\underset{m,n}{\sum }\left\{\left(i{\dot{\lambda }}_{b^{m}}^{j^{m}}+\langle \Psi_{\text{L}}|[Ĥ-i\hat{X},{Ê}_{j^{m}}^{b^{m}}]|\Psi_{\text{R}}\rangle \right)\right.\\ & \;\left.\times \langle \Phi_{j^{m}}^{b^{m}}|e^{-\hat{T}}(O_{k^{n}}^{i^{n}}{Ê}_{k^{n}}^{i^{n}}+O_{a^{n}}^{c^{n}}{Ê}_{a^{n}}^{c^{n}})|\Psi_{\text{R}}\rangle -c.c.\right\}\\ & \;+\underset{m,n}{\sum }\left\{\left(\langle \Phi_{j^{m}}^{b^{m}}|e^{-\hat{T}}(Ĥ-i\hat{X})|\Psi_{\text{R}}\rangle -i{\dot{\tau }}_{j^{m}}^{b^{m}}\right)\right.\\ & \;\left.\times \langle \Psi_{\text{L}}|[{Ê}_{j^{m}}^{b^{m}},(O_{k^{n}}^{i^{n}}{Ê}_{k^{n}}^{i^{n}}+O_{a^{n}}^{c^{n}}{Ê}_{a^{n}}^{c^{n}})]|\Psi_{\text{R}}\rangle -c.c.\right\}\;. \end{array} \end{array}$$

Here we assume Ô is Hermitian.

Equations obtained by the variation $\delta S_{\text{R}}$ and $\delta S_{\text{I}}$ with respect to $\Delta_{a^{m}}^{i^{m}}$ are

(39) $$\begin{array}{c} \begin{array}{cc} & 0=\langle \Psi_{\text{L}}|(Ĥ-i\hat{X})e^{\hat{T}}\bar{\hat{\Pi }}e^{-\hat{T}}{Ê}_{a^{m}}^{i^{m}}|\Psi_{\text{R}}\rangle \\ & \;-\langle \Psi_{\text{L}}|{Ê}_{a^{m}}^{i^{m}}e^{\hat{T}}\bar{\hat{\Pi }}e^{-\hat{T}}(Ĥ-i\hat{X})|\Psi_{\text{R}}\rangle \\ & \;+(i{\dot{\lambda }}_{b^{m}}^{j^{m}}+\langle \Psi_{\text{L}}|[Ĥ-i\hat{X},{Ê}_{j^{m}}^{b^{m}}]|\Psi_{\text{R}}\rangle )\langle \Phi_{j^{m}}^{b^{m}}|e^{-\hat{T}}{Ê}_{a^{m}}^{i^{m}}|\Psi_{\text{R}}\rangle \\ & \;+(\langle \Phi_{j^{m}}^{b^{m}}|e^{-\hat{T}}(Ĥ-i\hat{X})|\Psi_{\text{R}}\rangle -i{\dot{\tau }}_{j^{m}}^{b^{m}})\langle \Psi_{\text{L}}|[{Ê}_{j^{m}}^{b^{m}},{Ê}_{a^{m}}^{i^{m}}]|\Psi_{\text{R}}\rangle \\ & \;\mp ({\left\langle \Psi_{\text{L}}|[Ĥ-i\hat{X},{Ê}_{i^{m}}^{a^{m}}]|\Psi_{\text{R}}\right\rangle }^{*}-i{\left({\dot{\lambda }}_{a^{m}}^{i^{m}}\right)}^{*})\;, \end{array} \end{array}$$

where the minus (plus) sign applies to $\delta S_{\text{R}}$ ($\delta S_{\text{I}}$) in the last line. Therefore, as long as Equation (10) is selected, the Ehrenfest theorem is not violated by particle–hole rotations ($O_{a^{m}}^{i^{m}}$ and $O_{i^{m}}^{a^{m}}$ terms): they are NB5, NB8, NB11, NB14, NB16, and NB18, nonetheless, none of them satisfy the particle‐particle ($O_{a^{m}}^{b^{m}}$ terms) and hole‐hole ($O_{i^{m}}^{j^{m}}$ terms) rotation invariance. Therefore, none of NB methods satisfies the Ehrenfest theorem. This is a major difference between the ooCC methods and NB methods. In particular, even though NB1 to 3, NB 10, NB13, and NB15 are gauge invariant, they do not use Equation (10) as EOMs and thus still violate the Ehrenfest theorem.

### 3.6 Electron Dynamics in a Strong Laser Field

An important application of the TD‐ooCC is the electron dynamics in a strong laser field [15, 26]. For completeness, we follow the formulation detailed in our previous work Reference [26] to demonstrate the key difference between the TD‐ooCC and NB methods. A typical second quantized Hamiltonian of a many‐electron system in external semi‐classical electromagnetic fields under the electric dipole approximation is (fermion‐kind label is neglected)

(40) $$\begin{array}{ll} & \;\;\;\;Ĥ(t)=h_{\nu }^{\mu }(t){Ê}_{\nu }^{\mu }+\frac{1}{2}g_{\nu \delta }^{\mu \gamma }{Ê}_{\nu \delta }^{\mu \gamma },\; \end{array}$$

(41) $$\begin{array}{ll} & \;\;\;h_{\nu }^{\mu }(t)=\int dx\psi_{\mu }^{*}[h_{0}+V_{\text{ext}}(t)]\psi_{\nu },\; \end{array}$$

(42) $$\begin{array}{ll} & g_{\nu \delta }^{\mu \gamma }=\int \int dx_{1}dx_{2}\psi_{\mu }^{*}(x_{1})\psi_{\gamma }^{*}(x_{2})r_{12}^{-1}\psi_{\nu }(x_{1})\psi_{\delta }(x_{2}),\; \end{array}$$

where x=(r,σ) labels spatial‐spin coordinate, and $h_{0}$ is the gauge‐independent one‐electron field free Hamiltonian including the kinetic term and the electron‐fixed nuclei Coulomb's terms. $V_{\text{ext}}$ is the gauge‐dependent electron‐laser interaction term

(43) $$\begin{array}{cc} & \text{LG:}\;V_{\text{ext}}=E(t)\cdot r\;,\\ & \text{VG:}\;V_{\text{ext}}=-iA(t)\cdot \nabla_{r}\;,\\ & \;A(t)=-\int^{t}dt^{\prime }E(t^{\prime })\;. \end{array}$$

Simulation results in two gauges should be the same if the numerical convergence is achieved, while differences in convergence speed generally make the VG more suitable for strong‐field dynamics and the LG for other scenarios [74].

As discussed in the beginning of this section, all EOMs and constraints of orbital rotations of NB methods should be understood in the LG, and the non‐variance part of orbital rotations in the VG should be adjusted to ensure the gauge invariance as in Reference [47],

(44) $$X_{\mu }^{\nu }=iE(t)\cdot \langle \psi_{\nu }|r|\psi_{\mu }\rangle .$$

Gauge‐invariant NB methods [NB 1 to 3, NB10, NB13, and NB15] are expected to be more favorable than non‐invariant NB methods as well as TD‐OCCX0 in terms of the convergence of the grid points, because the evaluation of position operator matrix elements between relatively diffuse active orbitals is always avoided. However, the additional complexities of the modified Ehrenfest theorem in all NB methods often present more complications than in TD‐ooCC methods.

## 4 Conclusion and Outlook

In this work, we have systematically formulated the TD‐oqoCC‐NB family of methods for arbitrary fermion mixtures, and comprehensively investigated the mathematical structures and physical properties of these methods, especially the application to vibrational and electron dynamics. By selectively combining the EOMs for single CC amplitudes and particle‐hole orbital rotations without strictly adhering to the TD‐BIVP, we presented 18 distinct theoretical schemes (NB1 to NB18), and nine of them (NB10 to NB18) can converge to the TD‐CASSCF. Truncations of non‐single CC amplitudes and orbital rotations other than particle‐hole are identical to the TD‐ooCC. EOMs of NB2, NB4, NB7 to 10, and NB18 do not require the inverse of matrix composed of different modes, thus favorable for systems with large number of fermion modes. The major distinction between TD‐oqoCC‐NB and TD‐ooCC is that the former one cannot ensure the Ehrenfest theorem even though some of them [NB1 to 3, NB10, Nb13, and NB15] are gauge invariant. For electron dynamics in a strong laser field, one should use gauge invariant methods with the implementation of modified Ehrenfest theorems. We emphasize the recommendation lists here satisfy necessary properties and serve as a guide for future numerical benchmarking, and the optimal choices of methods should be benchmarked within the list. When dynamical cores and virtual orbitals do not exist, NB3 and NB15 are analogs of TD‐OCC and TD‐BCC based on the imaginary part action TD‐BIVP. Constraints in NB methods are expected to make algorithms numerically stable at finite truncations. We also discussed the potential instability of NB3 arisen from close to the degenerate manifold.

Similarly to TD‐ooCC, one can also use different TD‐oqoCC‐NB EOMs for different fermion kinds [26], yielding TD‐oqoCC‐NB‐hybrid methods. Beyond the application of NB methods in time‐dependent dynamics, they also serve as novel CC methods for time‐independent electronic structure calculations, in which the stationary equations are defined by setting all time derivatives in EOMs to zero.

## Funding

This work was supported by the Ministry of Education, Culture, Sports, Science and Technology (MEXT) of Japan (Grant No. JP22H05025 and JP25K01688), the MEXT Quantum Leap Flagship Program (MEXT Q‐LEAP) (Grant No. JPMXS0118067246), the JST COI‐NEXT (Grant No. JPMJPF2221), IBM‐UTokyo lab, and RIKEN TRIP initiative.

## Conflicts of Interest

The authors declare no conflicts to disclose.

## Data Availability

Data sharing is not applicable to this article as no new datasets were created or analyzed during the current study.

## Appendix A EOMs and Superoperators

### EOMs of Orbital Rotations

The expression in this subsection is mainly based on Reference [26]. In practical simulations, one always uses the explicit simplifications [75] of Equation (4),

(A1)
$$\begin{array}{ll} 2iX_{i^{\prime m}}^{u^{m}} & (\delta_{u^{m}}^{t^{m}}-D_{u^{m}}^{t^{m}})=\\ & \langle \Psi_{\text{L}}|{Ê}_{t^{m}}^{i^{\prime m}}Ĥ|\Psi_{\text{R}}\rangle +\langle \Psi_{\text{R}}|{Ê}_{t^{m}}^{i^{\prime m}}Ĥ|\Psi_{\text{L}}\rangle \;,\; \end{array}$$

(A2)
$$\begin{array}{ll} 2iX_{q^{m}}^{\alpha^{m}} & D_{p^{m}}^{q^{m}}=\langle \Psi_{\text{L}}|{Ê}_{\alpha^{m}}^{p^{m}}Ĥ|\Psi_{\text{R}}\rangle +\langle \Psi_{\text{R}}|{Ê}_{\alpha^{m}}^{p^{m}}Ĥ|\Psi_{\text{L}}\rangle \;,\; \end{array}$$

which are sufficient for non‐virtural orbital rotations. However, the absence of propagation of virtual orbitals forces us to convert EOMs to a more practical form [26, 41, 76], see equation (22) of Reference [26].

### Definitions of the Unified Superoperators and Sources

We first define the numerical kernels as

(A3)
$$\begin{array}{ll} R{\left(Ô\right)}_{i^{m}}^{a^{m}} & =\langle \Phi_{i^{m}}^{a^{m}}|e^{-\hat{T}}Ô|\Psi_{\text{R}}\rangle \;,\; \end{array}$$

(A4)
$$\begin{array}{ll} L{\left(Ô\right)}_{i^{m}}^{a^{m}} & =\langle \Psi_{\text{L}}|[Ô,{Ê}_{i^{m}}^{a^{m}}]|\Psi_{\text{R}}\rangle \;,\; \end{array}$$

(A5)
$$\begin{array}{ll} O_{1}{\left(Y\right)}_{i^{m}}^{a^{m}} & =\underset{n}{\sum }\langle \Phi_{i^{m}}^{a^{m}}|e^{-\hat{T}}{Ê}_{b^{n}}^{j^{n}}|\Psi_{\text{R}}\rangle Y_{b^{n}}^{j^{n}}\;,\; \end{array}$$

(A6)
$$\begin{array}{ll} {\tilde{O}}_{1}{\left(Y\right)}_{i^{m}}^{a^{m}} & =\underset{n}{\sum }\langle \Phi_{k^{n}}^{c^{n}}|e^{-\hat{T}}{Ê}_{a^{m}}^{i^{m}}|\Psi_{\text{R}}\rangle Y_{c^{n}}^{k^{n}}\;,\; \end{array}$$

(A7)
$$\begin{array}{ll} O_{2}{\left(X^{*}\right)}_{i^{m}}^{a^{m}} & =\underset{n}{\sum }A_{i^{m}b^{n}}^{a^{m}j^{n}}{\left(X_{j^{n}}^{b^{n}}\right)}^{*}\;,\; \end{array}$$

(A8)
$$\begin{array}{ll} M{\left(\Omega ,Y\right)}_{i^{m}}^{a^{m}} & =(\delta_{b^{m}}^{a^{m}}\Omega_{i^{m}}^{j^{m}}-\Omega_{b^{m}}^{a^{m}}\delta_{i^{m}}^{j^{m}})Y_{j^{m}}^{b^{m}}\;.\; \end{array}$$

The superoperators and source terms are defined as

(A9)
$$\begin{array}{ll} 𝕀[Y] & =Y\;,\; \end{array}$$

(A10)
$$\begin{array}{ll} {𝕀}_{O}[Y] & =Y+O_{1}(Y)\;,\; \end{array}$$

(A11)
$$\begin{array}{ll} {𝕄}_{\Omega }[Y] & =M(\Omega ,Y)\;(\Omega \in \{\rho ,\rho^{T},D,K\})\;,\; \end{array}$$

(A12)
$$\begin{array}{ll} {\tilde{𝕃}}_{\pm }[Y] & =\frac{1}{2}{\tilde{O}}_{1}(Y)\pm \frac{1}{2}Y^{*}\;,\; \end{array}$$

(A13)
$$\begin{array}{c} \begin{array}{ll} {𝕎}_{\pm }[X] & =\frac{1}{2}\left[O_{2}(X^{*})-M(\rho ,O_{1}(X))\pm M(\rho^{†},X)\right]\;,\; \end{array} \end{array}$$

(A14)
$$\begin{array}{c} \begin{array}{ll} 𝕌[X] & =\frac{1}{2}O_{2}(X^{*})+\frac{1}{2}M(\rho ,X)-\frac{1}{2}{\tilde{O}}_{1}(M(\rho^{T},X^{*}))\;.\; \end{array} \end{array}$$

(A15)
$$\begin{array}{ll} R_{0} & =R(Ĥ)\;,\;L_{0}=L(Ĥ)\;,\; \end{array}$$

(A16)
$$\begin{array}{ll} R_{X} & =R(Ĥ-i\hat{X})\;,\;L_{X}=L(Ĥ-i\hat{X})\;,\; \end{array}$$

(A17)
$$\begin{array}{ll} B_{D}[X] & =B-iM(D,X)-\frac{i}{2}O_{2}(X^{*})\;,\; \end{array}$$

(A18)
$$\begin{array}{ll} B_{K}[X] & =\tilde{B}-iM(K,X)-\frac{i}{2}O_{2}(X^{*})\;.\; \end{array}$$

All above superoperators and sources are defined in the LG. For the methods satisfying pp and hh rotation invariance, the EOMs in the LG and VG are formally the same. For the methods not satisfying pp and hh rotation invariance, $\hat{X}$ should be understood as $X_{i^{m}}^{a^{m}}{Ê}_{i^{m}}^{a^{m}}+X_{a^{m}}^{i^{m}}{Ê}_{a^{m}}^{i^{m}}$ and Ĥ should be understood as $Ĥ-iX_{i^{m}}^{j^{m}}{Ê}_{i^{m}}^{j^{m}}-iX_{a^{m}}^{b^{m}}{Ê}_{a^{m}}^{b^{m}}$ and the EOMs in the VG and LG are formally the same.

### Explicit EOMs of NB Methods

For completeness, we list all EOMs of NB methods explicitly here,

#### NB1

(A19)
$$\begin{array}{ll} iX_{i^{m}}^{a^{m}}+\underset{n}{\sum }i & \langle \Phi_{i^{m}}^{a^{m}}|e^{-\hat{T}}{Ê}_{b^{n}}^{j^{n}}|\Psi_{\text{R}}\rangle X_{b^{n}}^{j^{n}}=\langle \Phi_{i^{m}}^{a^{m}}|e^{-\hat{T}}Ĥ|\Psi_{\text{R}}\rangle \;,\; \end{array}$$

#### NB2

(A20)
$$i\left(\rho_{j^{m}}^{i^{m}}\delta_{b^{m}}^{a^{m}}-\rho_{a^{m}}^{b^{m}}\delta_{j^{m}}^{i^{m}}\right)X_{b^{m}}^{j^{m}}=\langle \Psi_{\text{L}}|[Ĥ,{Ê}_{i^{m}}^{a^{m}}]|\Psi_{\text{R}}\rangle ,$$

#### NB3

(A21)
$$\begin{array}{c} i(\delta_{b^{m}}^{a^{m}}K_{i^{m}}^{j^{m}}-K_{b^{m}}^{a^{m}}\delta_{i^{m}}^{j^{m}})X_{j^{m}}^{b^{m}}+i\frac{1}{2}\underset{n}{\sum }\left\{A_{i^{m}b^{n}}^{a^{m}j^{n}}{\left(X_{j^{n}}^{b^{n}}\right)}^{*}\right\}={\tilde{B}}_{i^{m}}^{a^{m}}\;, \end{array}$$

#### NB4

(A22)
$$-i{\dot{\lambda }}_{a^{m}}^{i^{m}}=\langle \Psi_{\text{L}}|[Ĥ-i\hat{X},{Ê}_{i^{m}}^{a^{m}}]|\Psi_{\text{R}}\rangle .$$

#### NB5

(A23)
$$\begin{array}{cc} & i\frac{1}{2}\underset{n}{\sum }\left\{\langle \Phi_{j^{n}}^{b^{n}}|e^{-\hat{T}}{Ê}_{a^{m}}^{i^{m}}|\Psi_{\text{R}}\rangle {\dot{\lambda }}_{b^{n}}^{j^{n}}\right\}+i\frac{1}{2}{\left({\dot{\lambda }}_{a^{m}}^{i^{m}}\right)}^{*}=\\ & B_{i^{m}}^{a^{m}}-i(\delta_{b^{m}}^{a^{m}}D_{i^{m}}^{j^{m}}-D_{b^{m}}^{a^{m}}\delta_{i^{m}}^{j^{m}})X_{j^{m}}^{b^{m}}-i\frac{1}{2}\underset{n}{\sum }A_{i^{m}b^{n}}^{a^{m}j^{n}}{\left(X_{j^{n}}^{b^{n}}\right)}^{*}\;, \end{array}$$

#### NB6

(A24)
$$\begin{array}{cc} & i\frac{1}{2}\underset{n}{\sum }\left\{\langle \Phi_{j^{n}}^{b^{n}}|e^{-\hat{T}}{Ê}_{a^{m}}^{i^{m}}|\Psi_{\text{R}}\rangle {\dot{\lambda }}_{b^{n}}^{j^{n}}\right\}-i\frac{1}{2}{\left({\dot{\lambda }}_{a^{m}}^{i^{m}}\right)}^{*}=\\ & {\tilde{B}}_{i^{m}}^{a^{m}}-i(\delta_{b^{m}}^{a^{m}}K_{i^{m}}^{j^{m}}-K_{b^{m}}^{a^{m}}\delta_{i^{m}}^{j^{m}})X_{j^{m}}^{b^{m}}-i\frac{1}{2}\underset{n}{\sum }A_{i^{m}b^{n}}^{a^{m}j^{n}}{\left(X_{j^{n}}^{b^{n}}\right)}^{*}\;, \end{array}$$

#### NB7

(A25)
$$\begin{array}{l} i{\dot{\tau }}_{i^{m}}^{a^{m}}=\langle \Phi_{i^{m}}^{a^{m}}|e^{-\hat{T}}(Ĥ-i\hat{X})|\Psi_{\text{R}}\rangle \;, \end{array}$$

#### NB8

(A26)
$$\begin{array}{cc} & \frac{i}{2}(\delta_{b^{m}}^{a^{m}}\rho_{i^{m}}^{j^{m}}-\rho_{b^{m}}^{a^{m}}\delta_{i^{m}}^{j^{m}}){\dot{\tau }}_{j^{m}}^{b^{m}}=B_{i^{m}}^{a^{m}}\\ & \;-i(\delta_{b^{m}}^{a^{m}}D_{i^{m}}^{j^{m}}-D_{b^{m}}^{a^{m}}\delta_{i^{m}}^{j^{m}})X_{j^{m}}^{b^{m}}-i\frac{1}{2}\underset{n}{\sum }A_{i^{m}b^{n}}^{a^{m}j^{n}}{\left(X_{j^{n}}^{b^{n}}\right)}^{*}\;, \end{array}$$

#### NB9

(A27)
$$\begin{array}{cc} & \frac{i}{2}(\delta_{b^{m}}^{a^{m}}\rho_{i^{m}}^{j^{m}}-\rho_{b^{m}}^{a^{m}}\delta_{i^{m}}^{j^{m}}){\dot{\tau }}_{j^{m}}^{b^{m}}={\tilde{B}}_{i^{m}}^{a^{m}}\\ & \;-i(\delta_{b^{m}}^{a^{m}}K_{i^{m}}^{j^{m}}-K_{b^{m}}^{a^{m}}\delta_{i^{m}}^{j^{m}})X_{j^{m}}^{b^{m}}-i\frac{1}{2}\underset{n}{\sum }A_{i^{m}b^{n}}^{a^{m}j^{n}}{\left(X_{j^{n}}^{b^{n}}\right)}^{*}\;, \end{array}$$

#### NB10

(A28)
$$\begin{array}{ll} & i\left(\rho_{j^{m}}^{i^{m}}\delta_{b^{m}}^{a^{m}}-\rho_{a^{m}}^{b^{m}}\delta_{j^{m}}^{i^{m}}\right)X_{b^{m}}^{j^{m}}=\langle \Psi_{\text{L}}|[Ĥ,{Ê}_{i^{m}}^{a^{m}}]|\Psi_{\text{R}}\rangle \;,\; \end{array}$$

(A29)
$$\begin{array}{ll} & i{\dot{\tau }}_{i^{m}}^{a^{m}}=\langle \Phi_{i^{m}}^{a^{m}}|e^{-\hat{T}}(Ĥ-i\hat{X})|\Psi_{\text{R}}\rangle \;,\; \end{array}$$

#### NB11

(A30)
$$\begin{array}{c} \begin{array}{cc} & i\frac{1}{2}\underset{n}{\sum }\left\{A_{i^{m}b^{n}}^{a^{m}j^{n}}+(\delta_{c^{m}}^{a^{m}}\rho_{i^{m}}^{k^{m}}-\rho_{c^{m}}^{a^{m}}\delta_{i^{m}}^{k^{m}})\langle \Phi_{k^{m}}^{c^{m}}|e^{-\hat{T}}{Ê}_{b^{n}}^{j^{n}}|\Psi_{\text{R}}\rangle \right\}{\left(X_{j^{n}}^{b^{n}}\right)}^{*}\\ & \;+\frac{i}{2}{\left(\delta_{b^{m}}^{a^{m}}\rho_{j^{m}}^{i^{m}}-\rho_{a^{m}}^{b^{m}}\delta_{i^{m}}^{j^{m}}\right)}^{*}X_{j^{m}}^{b^{m}}\\ & \;=B_{i^{m}}^{a^{m}}-\frac{1}{2}(\delta_{b^{m}}^{a^{m}}\rho_{i^{m}}^{j^{m}}-\rho_{b^{m}}^{a^{m}}\delta_{i^{m}}^{j^{m}})\langle \Phi_{j^{m}}^{b^{m}}|e^{-\hat{T}}Ĥ|\Psi_{\text{R}}\rangle \;, \end{array} \end{array}$$

(A31)
$$\begin{array}{ll} i{\dot{\tau }}_{i^{m}}^{a^{m}} & =\langle \Phi_{i^{m}}^{a^{m}}|e^{-\hat{T}}(Ĥ-i\hat{X})|\Psi_{\text{R}}\rangle \;,\; \end{array}$$

#### NB12

(A32)
$$\begin{array}{c} \begin{array}{cc} & i\frac{1}{2}\underset{n}{\sum }\left\{A_{i^{m}b^{n}}^{a^{m}j^{n}}+(\delta_{c^{m}}^{a^{m}}\rho_{i^{m}}^{k^{m}}-\rho_{c^{m}}^{a^{m}}\delta_{i^{m}}^{k^{m}})\langle \Phi_{k^{m}}^{c^{m}}|e^{-\hat{T}}{Ê}_{b^{n}}^{j^{n}}|\Psi_{\text{R}}\rangle \right\}{\left(X_{j^{n}}^{b^{n}}\right)}^{*}\\ & \;-\frac{i}{2}{\left(\delta_{b^{m}}^{a^{m}}\rho_{j^{m}}^{i^{m}}-\rho_{a^{m}}^{b^{m}}\delta_{i^{m}}^{j^{m}}\right)}^{*}X_{j^{m}}^{b^{m}}\\ & \;={\tilde{B}}_{i^{m}}^{a^{m}}-\frac{1}{2}(\delta_{b^{m}}^{a^{m}}\rho_{i^{m}}^{j^{m}}-\rho_{b^{m}}^{a^{m}}\delta_{i^{m}}^{j^{m}})\langle \Phi_{j^{m}}^{b^{m}}|e^{-\hat{T}}Ĥ|\Psi_{\text{R}}\rangle \;, \end{array} \end{array}$$

(A33)
$$\begin{array}{ll} i{\dot{\tau }}_{i^{m}}^{a^{m}} & =\langle \Phi_{i^{m}}^{a^{m}}|e^{-\hat{T}}(Ĥ-i\hat{X})|\Psi_{\text{R}}\rangle \;,\; \end{array}$$

#### NB13

(A34)
$$\begin{array}{ll} & iX_{i^{m}}^{a^{m}}+\underset{n}{\sum }i\langle \Phi_{i^{m}}^{a^{m}}|e^{-\hat{T}}{Ê}_{b^{n}}^{j^{n}}|\Psi_{\text{R}}\rangle X_{b^{n}}^{j^{n}}=\langle \Phi_{i^{m}}^{a^{m}}|e^{-\hat{T}}Ĥ|\Psi_{\text{R}}\rangle \;,\; \end{array}$$

(A35)
$$\begin{array}{ll} & \;-i{\dot{\lambda }}_{a^{m}}^{i^{m}}=\langle \Psi_{\text{L}}|[Ĥ-i\hat{X},{Ê}_{i^{m}}^{a^{m}}]|\Psi_{\text{R}}\rangle \;.\; \end{array}$$

#### NB14

(A36)
$$\begin{array}{cc} & i\frac{1}{2}\underset{n}{\sum }\left\{A_{i^{m}b^{n}}^{a^{m}j^{n}}{\left(X_{j^{n}}^{b^{n}}\right)}^{*}\right\}+\frac{i}{2}(\delta_{b^{m}}^{a^{m}}\rho_{i^{m}}^{j^{m}}-\rho_{b^{m}}^{a^{m}}\delta_{i^{m}}^{j^{m}})X_{j^{m}}^{b^{m}}\\ & -\frac{i}{2}\langle \Phi_{k^{n}}^{c^{n}}|e^{-\hat{T}}{Ê}_{a^{m}}^{i^{m}}|\Psi_{\text{R}}\rangle \left(\rho_{j^{n}}^{k^{n}}\delta_{b^{n}}^{c^{n}}-\rho_{c^{n}}^{b^{n}}\delta_{j^{n}}^{k^{n}}\right){\left(X_{j^{n}}^{b^{n}}\right)}^{*}=\\ & B_{i^{m}}^{a^{m}}+\frac{1}{2}\underset{n}{\sum }\left\{\left\langle \Phi_{j^{n}}^{b^{n}}|e^{-\hat{T}}{Ê}_{a^{m}}^{i^{m}}|\Psi_{\text{R}}\rangle \langle \Psi_{\text{L}}|[Ĥ,{Ê}_{j^{n}}^{b^{n}}]|\Psi_{\text{R}}\right\rangle \right\}\\ & \;-\frac{1}{2}{\left\langle \Psi_{\text{L}}|[Ĥ,{Ê}_{i^{m}}^{a^{m}}]|\Psi_{\text{R}}\right\rangle }^{*}\;, \end{array}$$

(A37)
$$\begin{array}{ll} -i{\dot{\lambda }}_{a^{m}}^{i^{m}} & =\langle \Psi_{\text{L}}|[Ĥ-i\hat{X},{Ê}_{i^{m}}^{a^{m}}]|\Psi_{\text{R}}\rangle \;.\; \end{array}$$

#### NB15

(A38)
$$\begin{array}{l} iX_{i^{m}}^{a^{m}}+\underset{n}{\sum }i\langle \Phi_{i^{m}}^{a^{m}}|e^{-\hat{T}}{Ê}_{b^{n}}^{j^{n}}|\Psi_{\text{R}}\rangle X_{b^{n}}^{j^{n}}=\langle \Phi_{i^{m}}^{a^{m}}|e^{-\hat{T}}Ĥ|\Psi_{\text{R}}\rangle \;, \end{array}$$

(A39)
$$\begin{array}{cc} & i\frac{1}{2}\underset{n}{\sum }\left\{\langle \Phi_{j^{n}}^{b^{n}}|e^{-\hat{T}}{Ê}_{a^{m}}^{i^{m}}|\Psi_{\text{R}}\rangle {\dot{\lambda }}_{b^{n}}^{j^{n}}\right\}-i\frac{1}{2}{\left({\dot{\lambda }}_{a^{m}}^{i^{m}}\right)}^{*}=\\ & {\tilde{B}}_{i^{m}}^{a^{m}}-i(\delta_{b^{m}}^{a^{m}}K_{i^{m}}^{j^{m}}-K_{b^{m}}^{a^{m}}\delta_{i^{m}}^{j^{m}})X_{j^{m}}^{b^{m}}-i\frac{1}{2}\underset{n}{\sum }A_{i^{m}b^{n}}^{a^{m}j^{n}}{\left(X_{j^{n}}^{b^{n}}\right)}^{*}\;, \end{array}$$

#### NB16

(A40)
$$\begin{array}{ll} i{\dot{\tau }}_{i^{m}}^{a^{m}} & =\langle \Phi_{i^{m}}^{a^{m}}|e^{-\hat{T}}(Ĥ-i\hat{X})|\Psi_{\text{R}}\rangle \;,\; \end{array}$$

(A41)
$$\begin{array}{cc} & i\frac{1}{2}\underset{n}{\sum }\left\{\langle \Phi_{j^{n}}^{b^{n}}|e^{-\hat{T}}{Ê}_{a^{m}}^{i^{m}}|\Psi_{\text{R}}\rangle {\dot{\lambda }}_{b^{n}}^{j^{n}}\right\}+i\frac{1}{2}{\left({\dot{\lambda }}_{a^{m}}^{i^{m}}\right)}^{*}=\\ & B_{i^{m}}^{a^{m}}-i(\delta_{b^{m}}^{a^{m}}D_{i^{m}}^{j^{m}}-D_{b^{m}}^{a^{m}}\delta_{i^{m}}^{j^{m}})X_{j^{m}}^{b^{m}}-i\frac{1}{2}\underset{n}{\sum }\left\{A_{i^{m}b^{n}}^{a^{m}j^{n}}{\left(X_{j^{n}}^{b^{n}}\right)}^{*}\right\}\\ & \;-\frac{i}{2}(\delta_{b^{m}}^{a^{m}}\rho_{i^{m}}^{j^{m}}-\rho_{b^{m}}^{a^{m}}\delta_{i^{m}}^{j^{m}}){\dot{\tau }}_{j^{m}}^{b^{m}}\;, \end{array}$$

#### NB17

(A42)
$$\begin{array}{ll} i{\dot{\tau }}_{i^{m}}^{a^{m}} & =\langle \Phi_{i^{m}}^{a^{m}}|e^{-\hat{T}}(Ĥ-i\hat{X})|\Psi_{\text{R}}\rangle \;,\; \end{array}$$

(A43)
$$\begin{array}{cc} & i\frac{1}{2}\underset{n}{\sum }\left\{\langle \Phi_{j^{n}}^{b^{n}}|e^{-\hat{T}}{Ê}_{a^{m}}^{i^{m}}|\Psi_{\text{R}}\rangle {\dot{\lambda }}_{b^{n}}^{j^{n}}\right\}-i\frac{1}{2}{\left({\dot{\lambda }}_{a^{m}}^{i^{m}}\right)}^{*}=\\ & {\tilde{B}}_{i^{m}}^{a^{m}}-i(\delta_{b^{m}}^{a^{m}}K_{i^{m}}^{j^{m}}-K_{b^{m}}^{a^{m}}\delta_{i^{m}}^{j^{m}})X_{j^{m}}^{b^{m}}-i\frac{1}{2}\underset{n}{\sum }\left\{A_{i^{m}b^{n}}^{a^{m}j^{n}}{\left(X_{j^{n}}^{b^{n}}\right)}^{*}\right\}\\ & \;-\frac{i}{2}(\delta_{b^{m}}^{a^{m}}\rho_{i^{m}}^{j^{m}}-\rho_{b^{m}}^{a^{m}}\delta_{i^{m}}^{j^{m}}){\dot{\tau }}_{j^{m}}^{b^{m}}\;, \end{array}$$

#### NB18

(A44)
$$\begin{array}{ll} -i{\dot{\lambda }}_{a^{m}}^{i^{m}} & =\langle \Psi_{\text{L}}|[Ĥ-i\hat{X},{Ê}_{i^{m}}^{a^{m}}]|\Psi_{\text{R}}\rangle \;.\; \end{array}$$

(A45)
$$\begin{array}{cc} & \frac{i}{2}(\delta_{b^{m}}^{a^{m}}\rho_{i^{m}}^{j^{m}}-\rho_{b^{m}}^{a^{m}}\delta_{i^{m}}^{j^{m}}){\dot{\tau }}_{j^{m}}^{b^{m}}=\\ & \;-i(\delta_{b^{m}}^{a^{m}}D_{i^{m}}^{j^{m}}-D_{b^{m}}^{a^{m}}\delta_{i^{m}}^{j^{m}})X_{j^{m}}^{b^{m}}-i\frac{1}{2}{\left({\dot{\lambda }}_{a^{m}}^{i^{m}}\right)}^{*}\\ & \;+B_{i^{m}}^{a^{m}}-i\frac{1}{2}\underset{n}{\sum }\left\{\langle \Phi_{j^{n}}^{b^{n}}|e^{-\hat{T}}{Ê}_{a^{m}}^{i^{m}}|\Psi_{\text{R}}\rangle {\dot{\lambda }}_{b^{n}}^{j^{n}}+A_{i^{m}b^{n}}^{a^{m}j^{n}}{\left(X_{j^{n}}^{b^{n}}\right)}^{*}\right\}\;, \end{array}$$
